# Diversity and Abundance of the Bacterial Community of the Red Macroalga *Porphyra umbilicalis*: Did Bacterial Farmers Produce Macroalgae?

**DOI:** 10.1371/journal.pone.0058269

**Published:** 2013-03-20

**Authors:** Lilibeth N. Miranda, Keith Hutchison, Arthur R. Grossman, Susan H. Brawley

**Affiliations:** 1 School of Marine Sciences, University of Maine, Orono, Maine, United States of America; 2 Department of Molecular and Biomedical Sciences, University of Maine, Orono, Maine, United States of America; 3 Carnegie Institution for Science, Department of Plant Biology, Stanford, California, United States of America; University of Waterloo, Canada

## Abstract

Macroalgae harbor microbial communities whose bacterial biodiversity remains largely uncharacterized. The goals of this study were 1) to examine the composition of the bacterial community associated with *Porphyra umbilicalis* Kützing from Schoodic Point, ME, 2) determine whether there are seasonal trends in species diversity but a core group of bacteria that are always present, and 3) to determine how the microbial community associated with a laboratory strain (P.um.1) established in the presence of antibiotics has changed. *P. umbilicalis* blades (n = 5, fall 2010; n = 5, winter 2011; n = 2, clonal P.um.1) were analyzed by pyrosequencing over two variable regions of the 16 S rDNA (V5–V6 and V8; 147,880 total reads). The bacterial taxa present were classified at an 80% confidence threshold into eight phyla (Bacteroidetes, Proteobacteria, Planctomycetes, Chloroflexi, Actinobacteria, Deinococcus-Thermus, Firmicutes, and the candidate division TM7). The Bacteroidetes comprised the majority of bacterial sequences on both field and lab blades, but the Proteobacteria (Alphaproteobacteria, Gammaproteobacteria) were also abundant. Sphingobacteria (Bacteroidetes) and Flavobacteria (Bacteroidetes) had inverse abundances on natural versus P.um.1 blades. Bacterial communities were richer and more diverse on blades sampled in fall compared to winter. Significant differences were observed between microbial communities among all three groups of blades examined. Only two OTUs were found on all 12 blades, and only one of these, belonging to the Saprospiraceae (Bacteroidetes), was abundant. *Lewinella* (as 66 OTUs) was found on all field blades and was the most abundant genus. Bacteria from the Bacteroidetes, Proteobacteria and Planctomycetes that are known to digest the galactan sulfates of red algal cell walls were well-represented. Some of these taxa likely provide essential morphogenetic and beneficial nutritive factors to *P. umbilicalis* and may have had unexpected effects upon evolution of macroalgal form as well as function.

## Introduction

Algal-bacterial associations are important to the biology of both groups of organisms, but the biodiversity of these associations remains largely unexplored and the ways in which bacterial-algal interactions impact metabolic and developmental features of the partner organisms are not well understood. A range of symbiotic, pathological and opportunistic interactions between macroalgae and bacteria are being discovered [1]. Depending upon the membership of its bacterial biofilm, a macroalga may suffer serious disease (e.g., [1]–[4]) or be protected from damaging colonization (e.g., [1], [5]). Algal defensive strategies against bacteria range from cell wall sloughing to the release of diverse anti-microbial compounds ([1], [6] e.g., hydrogen peroxide and other oxidative burst responses [7], [8], furanones that block bacterial communication [3]). The clear symbiotic associations between some algae and some bacteria include bacterial supply of vitamins, fixed nitrogen, and growth regulators to the algae, which, in turn, supply the bacteria with fixed carbon [9]. Provasoli and Pintner [10]–[12] were pioneers in understanding the obligatory nature of some bacterial-algal associations by discovering that normal algal morphology (e.g., of *Ulva* spp. and related green algae) was lost in axenic cultures. Normal algal morphology could sometimes be restored by reinfection with mixed bacterial cultures or by the application of plant hormones [12]–[14]. Many Bacteroidetes as well as some Proteobacteria and Firmicutes were found to support normal morphology when grown in association with ulvalean green algae (e.g., [15]–[17]), and “ thallusin” was isolated from a *Cytophaga* sp. (Bacteroidetes), crystallized, and demonstrated to be effective in restoring normal morphology in axenic cultures of ulvalean algae [18]. A few Bacteroidetes have just been found to induce multicellularity in choanoflagellates, close relatives of animals, by production of a sulfonolipid morphogen named RIF-1 [19]. Bacterial associations can be expected to be essential to *Porphyra umbilicalis,* because the blade phase of its close relative [20], *Pyropia yezoensis*, does not develop in axenic culture [21]).

Few studies have attempted to characterize the microbial communities of macroalgae with techniques that surmount the well-known under-sampling of communities by culture techniques. Studies at appropriate geographic scales with 16 S rDNA DGGE, 16 S rDNA clone libraries, and more recent metagenomic approaches have expanded our understanding of microbial communities associated with *Ulva* spp. [22]–[25], a few brown algae (e.g., *Fucus vesiculosus, Saccharina latissima* [25]–[26]) and a few red algae (e.g., *Delisea pulchra*, *Gracilaria vermiculophylla* [22], [25]). These studies have begun to address the scales of variation of the microbial community in space and time and have described differences related to the macroalgal host's taxonomy. Some investigators have also questioned whether there is a taxonomically-specific core microbiome on an algal host or only a constant core of microbial gene activities that can be provided by different sets of microbial taxa on the algal host [27]. These questions require additional studies, at greater sampling depth, on more algae, such as can be provided by pyrosequencing. Pyrosequencing of the variable regions of the 16 S rDNA [28] has transformed microbial ecology and is providing novel information about many natural and anthropogenic habitats [29]–[31]. Its recent application to microbial communities of several types of coral reef algae found different microbial communities on different algal genera and suggested that coral disease is being elevated by the increase in fleshy algal-associated bacteria in reef environments [32]–[33].

Here we describe the bacterial community associated with *P. umbilicalis* by high-throughput pyrosequencing of the 16 S rDNA. This alga is commercially important as human food (“laver”), and it is widely distributed on rocky intertidal shores of the north Atlantic [34]–[37]. Existing data on microbes of *Porphyra* sensu lato (i.e., including *Pyropia yezoensis*, “nori”) are limited to culture and cloning studies or intensive analysis of a few pathogenic bacteria belonging to Flavobacteria and Gammaproteobacteria ([38]–[46]). It is clear, however, that there is potential for discovery of much new microbial biodiversity associated with this group of algae, as demonstrated by the recent description of the class Phycisphaerae (Planctomycetes) from a *Porphyra* sp. [47]. The purpose of our studies was to describe the natural community on blades (gametophyes) in the intertidal zone at two different times, to compare the natural community to that remaining on clonal, antibiotic-treated blades that grow normally in laboratory culture, and to discover whether a core microbiome exists over the three conditions.

## Methods

### Ethics Statement

The scientific research and collecting permits were obtained for the described field studies from the United States Department of the Interior, National Park Service, Acadia National Park (permit #s: ACAD-2008, 2009, 2010, 2011-SCI-0004).

### Field samples

*Porphyra umbilicalis* was collected from the intertidal zone at Schoodic Point, Acadia National Park, Maine (44° 20′ 1.68" N, 68° 3′ 29.14" W) on October 28, 2010 (fall) and January 3, 2011 (winter). Five *P. umbilicalis* blades were collected individually in plastic bags using random numbers' sampling along a 30 m transect placed through the middle of the intertidal zone in each season. Positions for fall blades along the transect were 0 m (F1), 3 m (F2), 5 m (F3), 7 m (F4), 13 m (F5); winter blades were collected at 3 m (W1), 10 m (W2), 16 m (W3), 26 m (W4) and 28 m (W5). Blades were transported back to the laboratory on ice, rinsed briefly in 15°C autoclaved seawater within 2 h of collection to remove any superficially-attached microbes, frozen in liquid nitrogen, and stored at −80°C. The *P. umbilicalis* blade samples that were collected in the winter had fewer epiphytes based on observation with bright-field microscopy before blades were frozen.

### Laboratory samples

*Porphyra umbilicalis* laboratory blades were derived originally from a single blade collected at Schoodic Point on April 3, 2008; this is the same site where the field samples for this study were collected. Laboratory cultures in WES-PES seawater [48] were initially and repeatedly treated (10× over 2 years, 3 d each) with 100 mg L^−1^ penicillin G and 25 mg L^−1^ dihydrostreptomycin (1× PenStrep [48]) to reduce/eliminate bacteria. Blades for this study were part of the 7^th^ generation of the original isolate. Filtered aeration was provided by airstones, and the *P. umbilicalis* blades for this study were grown to maturity by attaching them to the side of transparent Nalgene containers (3 L) using aquarium seaweed clips. The cultures were incubated in Percival 136LL VL incubators (Percival Scientific, Inc., Perry, IA) at 10°C, a 14:10 L:D photoperiod and irradiance of 60 µmol photon m^−2^ s^−1^. Two clonal blades (hereafter “Lab”) were frozen and stored for DNA extraction. These blades experienced identical laboratory growth conditions and reproduction in the laboratory was exclusively by the generation of neutral spores [37], which makes the blades clonal. This is the isolate (“P.um.1”, UTEX LB2951) that is the subject of a JGI whole-genome sequencing project on *P. umbilicalis* [49].

### Genomic DNA isolation and amplification

Each sample consisted of an entire blade, allowing recovery of bacteria living on or inside any part of the blade. Each blade was ground in liquid nitrogen using a sterile mortar and pestle. About a 200 µl volume of the powdered tissue was transferred into an 1.5 ml microcentrifuge tube containing lysis buffer and RNase A solution. Tubes were placed on a rotator for 12 min at room temperature, and then genomic DNA was extracted using the DNeasy® Plant Mini Kit (Qiagen, Valencia, CA).

To explore the diversity of bacteria associated with *P. umbilicalis*, we obtained sequences of the V5–V7 and V8–V9 regions of the 16 S rRNA gene. Whole blade material was used for DNA extraction to minimize the potential loss of bacteria living inside blades, but this also leads to the extraction of chloroplast 16 S rDNA, which would be amplified by most primers that amplify bacterial 16 S rDNA sequences. To overcome this problem, we used a modified primer set 799f [5′-AMCVGGATTAGATACCCBG] [50] and 1492 r [5′-GYTACCTTGTTACGACTT] [51] that amplifies most bacterial sequences while excluding amplification of chloroplast 16 S rDNA [50]. PCR reactions were performed using the GoTaq® Green Master Mix (Promega, Madison, WI) in a 25 µl total volume (about 80 ng genomic DNA as template, 1 µl each of 10 µM forward and reverse primers, 9.5 µl sterile water and 12.5 µl GoTaq premix). PCR was performed using the following protocol: 94°C for 2 min followed by 30 cycles of 20 s at 94°C (denaturation), 40 s at 53°C (annealing), and 40 s at 72°C (extension), with a final extension at 72°C for 7 min. Two products of different size were obtained: a mitochondrial product (ca. 1100 bp in length) and a bacterial product (ca. 750 bp). The bacterial product was excised from the agarose gel and recovered using the illustra™ DNA and Gel Band Purification Kit (GE Healthcare, Piscataway, NJ). Because species diversity in a microbial assemblage from the same sample can vary due to different variable regions used in analysis [52]–[54], we compared the bacterial composition during fall 2010 and winter 2011 using two primer sets that recovered different hypervariable regions of the 16 S rDNA. The 750 bp PCR product was used as template for a second round of PCR following the same program as above, with the primer pairs 799 f and 1192 r (5′-CRTCCMCACCTTCCTC), for the V5–V7 region, and 1192 f (5′-GAGGAAGGTGKGGAYG) -1492 r for the V8-V9 region. The PCR products were purified using the DNA Clean and Concentrator™ -5 kit (Zymo Research, Irvine, CA) prior to 454 sequencing.

### Bacterial Tag-Encoded FLX 454-Pyrosequencing

Bacterial tag-encoded FLX 16 S rDNA amplicon pyrosequencing (bTEFAP) [55] was conducted by the Research and Testing Laboratory (RTL; Lubbock, TX) using the Genome Sequencer FLX instrument with Titanium protocols and reagents (Roche Applied Science, Indianapolis, IN). All PCR products used as sequencing templates were adjusted to 20 ng µl^−1^. Two pyrotag sequence libraries were generated by RTL from each of the 12 *Porphyra* blades using the primers given above, with barcoded sequences in each library recovered by sequencing from V5 through V7 or from V8 through V9. Raw sff files were provided by RTL for analysis, and these are available in the Sequence Read Archive (SRA, www.ncbi.nlm.nih.gov) under accession SRA065667/SRP018227. We used the mothur (v.1.27.0) software package [56] for curation and analysis of sequences. Mothur's implementation of PyroNoise was used with a default of 450 flows in denoising, and sequences were trimmed to a minimum length of 200 bp with default parameters in mothur. Sequences were aligned against the silva database in mothur, filtered, trimmed, and pre-clustered if they were within 2 bases of a more abundant sequence. Chimeras were detected with mothur's implementation of uchime, using each library as the reference set. The Gold reference database as an option in chimera.uchime (mothur.org) often works best for microbial communities that are well-studied, and it identified only about a third of the chimeric sequences found by using the *P. umbilicalis* pyrosequencing data as an internal reference to detect chimeras with the chimera.uchime command [57]. Sequences identified as being chimeric in the 12 samples in each dataset (V5V7: 39.6%+/−8.4% [mean, SD]; V8V9: 33.2%+/−5.5% [mean, SD]) were removed using the mothur pipeline.

Curation of the V5V7 sequences to reduce pyrosequencing errors resulted in a dataset in which sequences had a mean length of 266 bases (range: 249–281). Sequences began near *E. coli* position 800 [58] and ended near position 1066; this dataset thus begins in the conserved region preceding V5 and extends through variable region V6 to end in the conserved region forward of the V7; this dataset is referred to in the Results as the V5V6 dataset. Curation of the V8V9 sequences produced a dataset in which sequence mean length was 222 bases (range 197–232 bases) beginning near *E. coli* position 1190 and ending near position 1407. This means that these sequences include most of the conserved region before V8 and end just prior to the V9 region; this dataset is referred to as V8 in the rest of this study.

### Taxonomic classification, estimation of community richness and statistical analysis

Sequences were classified using the Ribosomal Database Project's rdp9 reference set in the mothur pipeline at an 80% confidence level, which is regarded as 89% accurate in classification at the genus level [59]. Operational taxonomic units (OTUs) were formed at 0.03, 0.05 and 0.10 distances, which are generally considered to correspond to species-, genus- and family-level classifications, using the average neighbor algorithm in mothur. We present alpha and beta diversity metrics for V8 samples before and after normalization (i.e., normalized in mothur to the V8 sample with the lowest number of sequences); however, we confine our analyses of V5V6 to taxon discovery in support of the V8 data (see Results and Discussion). A large proportion of sequences in the V5V6 library remained unclassified below the domain Bacteria (Table 1), and these were asymmetrically distributed among samples.

**Table 1:** Statistics for the sequences retrieved from the 16 S rDNA over the V5–V6 and V8 variable regions.

**A. Total sequences**		
	**V5–V6**	**V8**
Total sequences	106,366	41,514
Classified Bacteria	79,532	37,671
Unclassified Bacteria	26,834	3,843
		
**B. Total sequences by sample (plastid sequences removed**		
	**V5V6**	**V8**
Fall blade 1	8154	2613
Fall blade 2	3516	3864
Fall blade 3	10,296	2438
Fall blade 4	8405	2936
Fall blade 5	7393	2863
Winter blade 1	7875	4362
Winter blade 2	2658	3771
Winter blade 3	2581	4279
Winter blade 4	18129	4874
Winter blade 5	22790	5397
Lab blade 1	8210	2174
Lab blade 2	6359	1943
Total	106,366	41,514

Total sequences are those recovered after quality curation (denoising, trimming, chimera removal, and removal of *P. umbilicalis* chloroplast sequences).

Rarefaction analysis in mothur was used to examine the adequacy of the sampling depth for OTUs formed at 0.03 distance. V8 samples were rarefied by group over number of samples obtained for fall (n = 5), winter (n = 5) and lab (n = 2). V8 samples were normalized to the number of sequences in the smallest sample (L2, n = 1943 sequences, Table 1). Alpha-diversity was studied in the curated samples before and after normalization. The observed richness of samples at 0.03, 0.05, and 0.10 distance (i.e., 97%, 95%, 90% sequence similarity) was compared to the minimum predicted richness calculated by the Chao1 estimator in mothur. Diversity was examined by the inverse Simpson index. The microbiome was further analyzed by identifying OTUs formed at 0.03 distance that held ≥1% of sequences in the V8 and V5V6 samples, and OTUs (rare or abundant) that were shared over at least one sample of each of the three groups (fall, winter, lab) were recovered with the Venn command in mothur. The UPGMA algorithm using distance as calculated with the Jaccard coefficient was used to examine the dissimilarity of samples by membership with a dendrogram of the normalized V8 subsample while a distance matrix calculated using the Yue-Clayton theta similarity coefficient was used to produce a dendrogram that describes the samples based on abundance and membership. Dendrogram structure was tested for statistical significance with a weighted unifrac test in mothur. Thetayc (based on membership and abundance of OTUs) and Jaccard (based on membership) distance matrices were used in Analysis of Molecular Variance (AMOVA) to test whether the variation among fall, winter and lab normalized V8 samples was greater than variation between samples within each group. The level of significance for each experiment's AMOVA tests was set at 0.0125 (4 tests in each experiment). Biplots of OTUs significantly (*p*-value ≤0.05) associated with the axes of the non-metric dimensional scaling (nmds) ordination used to plot relationships of the 12 samples were plotted using the statistical computing package R (v.2.5.1[60]), including the vegan package (v. 2.0–5, [61]). Axis 1-axis 3 plots from a 3-dimensional nmds plot (R = 0.99, stress = 0.0399) are shown; calibrations with 3 abundant OTUs that were found only in winter or fall or on lab blades showed that OTUs significantly associated with axis 3 show affinity for the winter group; OTU vectors that point away from the axis 1 label have lab group affinities; OTU vectors that point toward the axis 1 label in the biplot graph have fall affinities.

We obtained relevant environmental data for the Maine coast close to our study site from a coastal GoMOOS environmental station (http://www.neracoos.org/gomoos; Accessed 2012 April 16) from October 1–31, 2010 (fall) and December 1–31, 2010 (winter). Photoperiod was estimated from the *Eldridge Tide and Pilot Book 2012* [62]. Although our analysis of the fall and winter blades likely shows seasonal trends, we emphasize that an additional year of sampling in each season would be required to confirm them as seasonal differences, as opposed to two different time points, because for logistical reasons, sampling was performed only once per season.

## Results

### Classification of the reads

A total of 106,366 (V5V6) and 41,514 (V8) sequences (Table 1) were obtained for analysis from raw reads following curation that included denoising, trimming, identification and removal of chimeras (see Methods), and removal of a small number of chloroplast sequences. Sequences assigned to the Cyanobacteria_Chloroplast category by the rdp9 database in mothur were all identified as *Porphyra umbilicalis* chloroplast sequences (best BLAST hit, followed by hits to four other bangialean plastids) and removed (1.1% [V5V6] and 0.3% [V8–V9] of sequences before this final curation) from trimmed and chimera-checked sequences before tabulation of curated sequences (Table 1). Most of the curated sequences (V5V6: 74.8%; V8: 90.7%) were classified to a particular bacterial phylum and class (or lower taxon). The proportion (25.2%, V5V6; 9.3%, V8) of sequences that were unclassified below the level of Bacteria (Table 1) probably represents a mixture of true biodiversity that was not classified as well as complex chimeras that were not identified by chimera.uchime (see Methods). Some V5V6 samples were dominated by these sequences, however, and we limit our use of V5V6 data to obtain information on the core microbiome and taxon memberships of the classified Bacteria (Table 1).

### Taxonomic overview of bacterial communities on Porphyra umbilicalis

**Figure 1:**
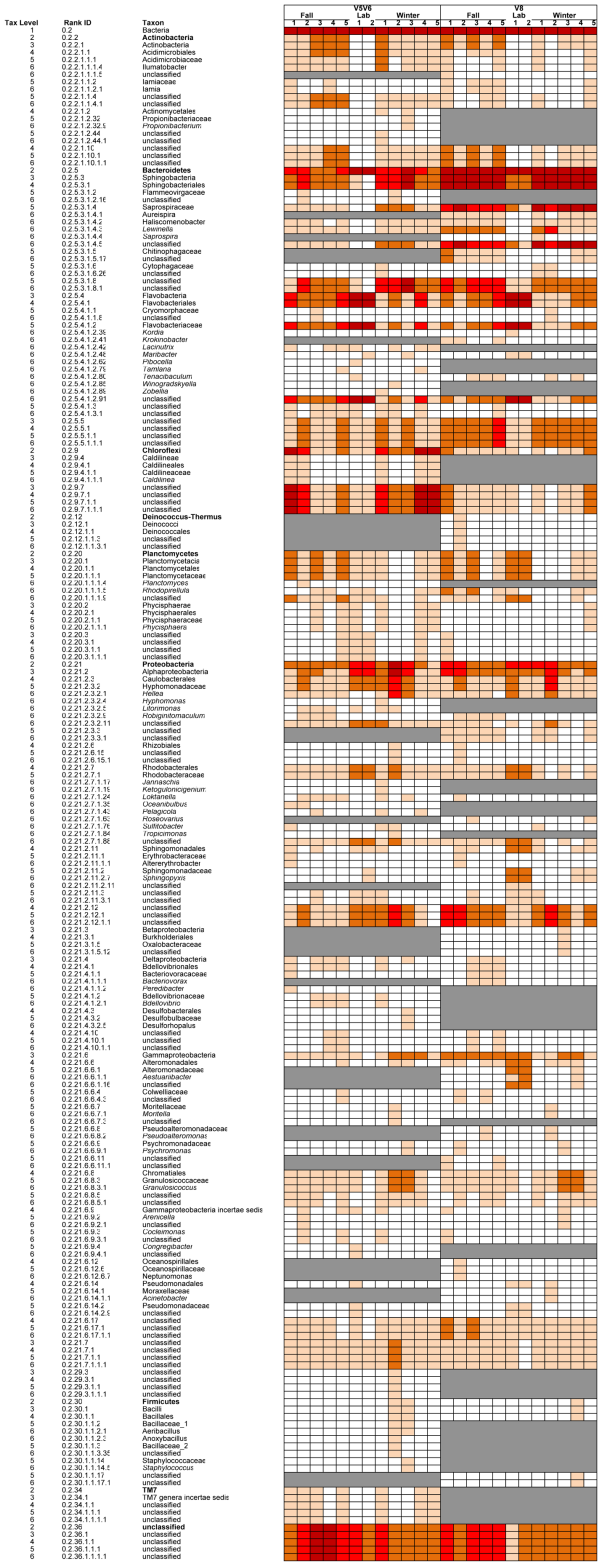
Taxonomic classification of sequences on individual blades of *Porphyra umbilicalis* (% abundance normalized to the number of sequences in each sample) using sequences recovered from both V8 and V5V6 libraries. Color codes are grey for absence in all blades; white for absence when the taxon was recovered in at least one blade (n = 12), light pink = >0 to 1%; orange  = >1% to 10%; red  = >10% to 50%; and dark red  = >50%. These categories make it possible to compare blades across libraries, but strictly the colors apply only vertically within each sample. The taxonomic level refers to phylum (2), class (3), order (4), family (5) and genus (6) here and in the supplemental tables.

**Figure 2:**
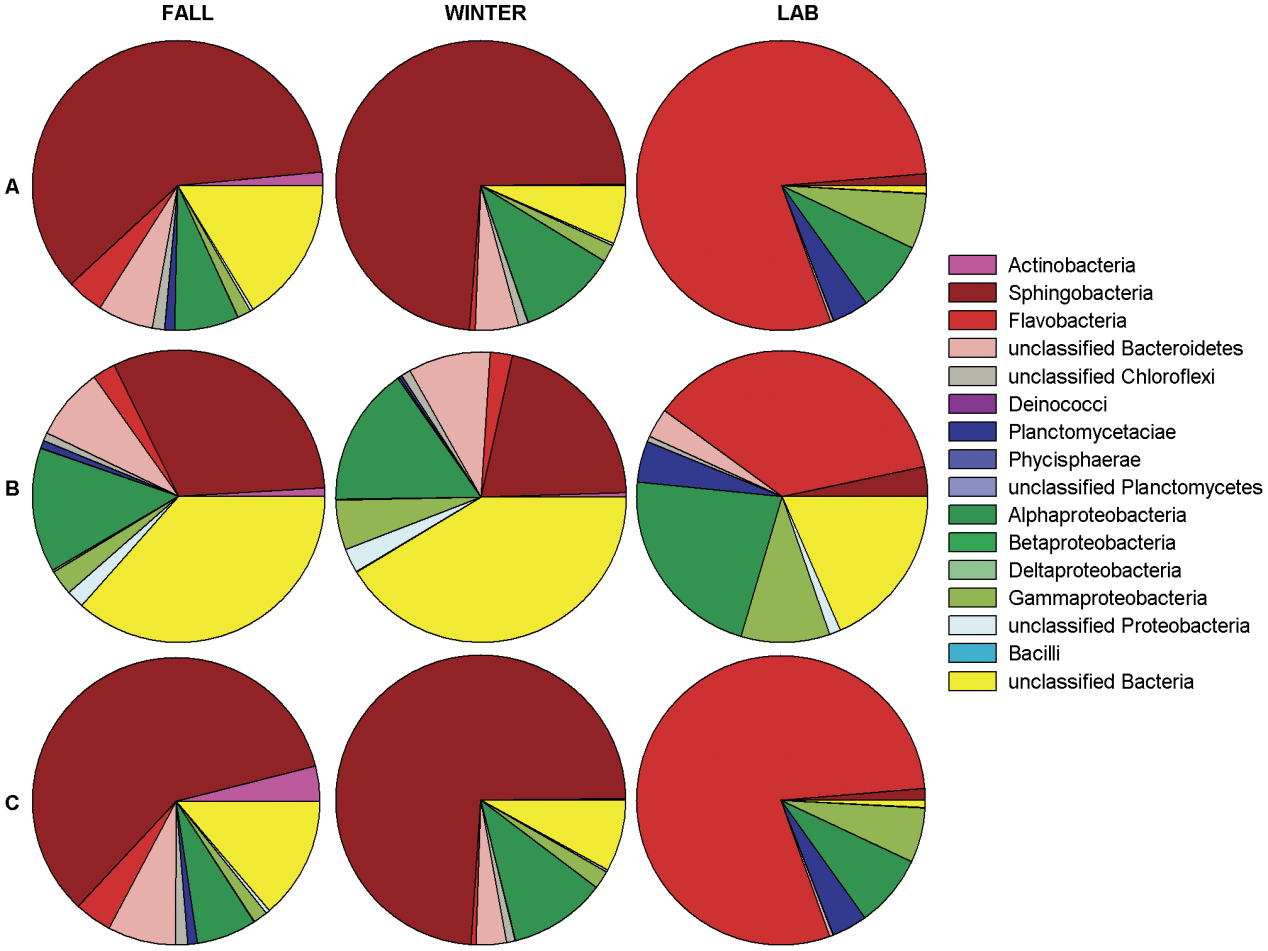
Bacterial community recovered in the V8 library from *Porphyra umbilicalis* in fall (n = 5 blades), winter (n = 5 blades) and lab P.um.1 (n = 2 blades). Pie-charts for the mean proportion of A) sequences, B) OTUs (0.03 distance), and C) sequences in normalized samples that belong to particular phyla and classes.

Seven phyla (Bacteroidetes, Proteobacteria, Actinobacteria, Chloroflexi, Planctomycetes, Firmicutes, Deinococcus-Thermus) were identified in the V8 library (Figs 1, 2; Table S1 in File S1). The classes Sphingobacteria (Bacteroidetes) and Alphaproteobacteria (Proteobacteria) dominated field blades in the V8 library both in overall sequence abundance and in the number of OTUs formed at 0.03 distance (Figs 1, 2; Tables S1,S2,S3 in File S1). The V5V6 library identified 6 of the 7 same phyla in field blades, but TM7 was found, whereas Deinococcus-Thermus (Fig.1) was not. Both of these phyla were identified at trace levels from the respective libraries. Proportional assignment of sequences to different phyla in the V5V6 library was similar to V8 except for a potentially equal abundance (but high variability among blades) of unclassified Chloroflexi compared to Bacteroidetes (Fig. 1). The penicilin-streptomycin treated, clonal laboratory blades also had a high proportion of Bacteroidetes but the proportions of Sphingobacteria (1%) compared to Flavobacteria (75%) on lab blades were close to inverse proportions of these classes on field blades (Figs. 1, 2; Tables S1, S2 in File S1). Alphaproteobacteria and Gammaproteobacteria were also common on lab blades whereas Actinobacteria, Deinococcus-Thermus, Firmicutes and TM7 appeared absent, no Betaproteobacteria or Deltaproteobacteria were found, and Chloroflexi were rare within both V8 and V5V6 libraries on laboratory blades (Fig. 1, Tables S1, S2 in File S1). The laboratory blades completed their normal [37] northwestern Atlantic life history (i.e., reproduction solely by neutral spores) with a generation time as short as 64 days at the time of sampling of lab blades for this study.

Sixty-one genera were found within the V8 library among the classified bacterial sequences, and 76 genera were found within the V5V6 library. Forty-eight of the genera recovered from the V8 and V5V6 sequences represent known (named) taxa (Table 2). Most of these were members of the Saprospiraceae (Sphingobacteria), Flavobacteria, Alphaproteobacteria and Gammaproteobacteria. Lab blades lacked 26 of these (Fig. 1, Table 2). A significant proportion of sequences remained unclassified at lower taxonomic levels, including within the Saprospiraceae and Cytophagaceae (Sphingobacteria) and Flavobacteriales (Flavobacteria) of the Bacteroidetes, the Chloroflexi, the Sphingomonadales (Alphaproteobacteria), and within the unclassified Bacteria (Fig. 1, Tables S2–S3 in File S1). *Lewinella* (Saprospiraceae) was particularly abundant on *P. umbilicalis* blades, representing a total of 3.35% of all sequences recovered in the V8 library (all from field samples), which clustered into 66 OTUs, by far the most OTUs within a described genus (Tables S2–S3 in File S1).

**Table 2:** Taxonomic summary of named genera found in the V5V6 or V8 libraries (I) and taxa found in only one library (V5V6, II; V8, III).

**I. Genera recovered by V5V6 or V8 tags**	
Actinobacteria (Actinobacteria):	*Ilumatobacter* *
	*Iamia* *
	*Propionibacterium* *
Sphingobacteria (Bacteroidetes)	*Aureispira* *
	*Haliscomenobacter* *
	*Lewinella* *
	*Saprospira* *
Flavobacteria (Bacteroidetes)	*Aureispira* *
	*Haliscomenobacter* *
	*Lewinella* *
	*Saprospira* *
	*Maribacter*
	*Pibocella*
	*Tamlana*
	*Tenacibaculum*
	*Winogradskyella*
	*Zobellia*
Caldilinea (Chloroflexi)	*Caldilinea* *
Deinococci (Deinococcus-Thermus)	(all unclassified)*
Planctomycetacia (Planctomycetes)	*Planctomyces* *
	*Rhodopirellula*
Phycisphaerae (Planctomycetes)	*Phycisphaera*
Alphaproteobacteria (Proteobacteria)	*Hellea*
	*Hyphomonas*
	*Litorimonas*
	*Robiginitomaculum* *
	*Jannaschia* *
	*Ketogulonicigenium* *
	*Loktanella* *
	*Oceanibulbus* *
	*Pelagicola*
	*Sulfitobacter*
	*Tropicimonas*
	*Sphingopyxis*
Deltaproteobacteria (Proteobacteria)	*Bacteriovorax* *
	*Peredibacter* *
	*Bdellovibrio* *
	*Desulforhopalus* *
Gammaproteobacteria (Proteobacteria)	*Aestuariibacter*
	*Pseudoalteromonas* *
	*Psychromonas* *
	*Granulosicoccus*
	*Arenicella*
	*Cocleimonas* *
	*Congregibacter*
	*Neptunomonas* *
	*Acinetobacter* *
Bacilli (Firmicutes)	*Aeribacillus* *
	*Anoxybacillus* *
	*Staphylococcus* *
TM7	(unclassified)*
**II. Taxa found by V5V6 and not V8**	
Actinobacteria	*Propionibacterium*
	*Lacinutrix*
	*Pibocella*
Flavobacteria (Bacteroidetes)	*Tamlana*
	*Winogradskyella*
	*Zobellia*
Chloroflexi	*Caldilinea*
Planctomycetes	*Planctomyces*
Alphaproteobacteria (Proteobacteria)	*Jannaschia*
	*Ketogulonicigenium*
	*Oceanibulbus*
	*Pelagicola*
	*Tropicimonas*
Deltaproteobacteria (Proteobacteria)	(none recovered in V8)
	*Peredibacter*
	*Bdellovibrio*
	*Desulforhopalus*
Gammaproteobacteria (Proteobacteria)	*Alteromonadaceae*
	*Aestuariibacter*
	*Congregibacter*
TM7	(none recovered in V8)
**III. Taxa found by V8 but not V5V6**	
Sphingobacteria (Bacteroidetes)	*Auresipira*
	*Saprospira*
	*Krokinobacter*
Deinococcus-Thermus	(none recovered in V5V6)
Alphaproteobacteria (Proteobacteria)	*Roseovarius* (Alphaproteobacteria)
Betaproteobacteria (Proteobacteria)	(none recovered in V5V6)
Deltaproteobacteria (Proteobacteria)	*Bacteriovorax*
Gammaproteobacteria (Proteobacteria)	*Pseudoalteromonas*
	*Neptunomonas*
	*Acinetobacter*

* Not present in laboratory blades

### Alpha-diversity metrics of microbial blade communities (V8 tags)

**Figure 3:**
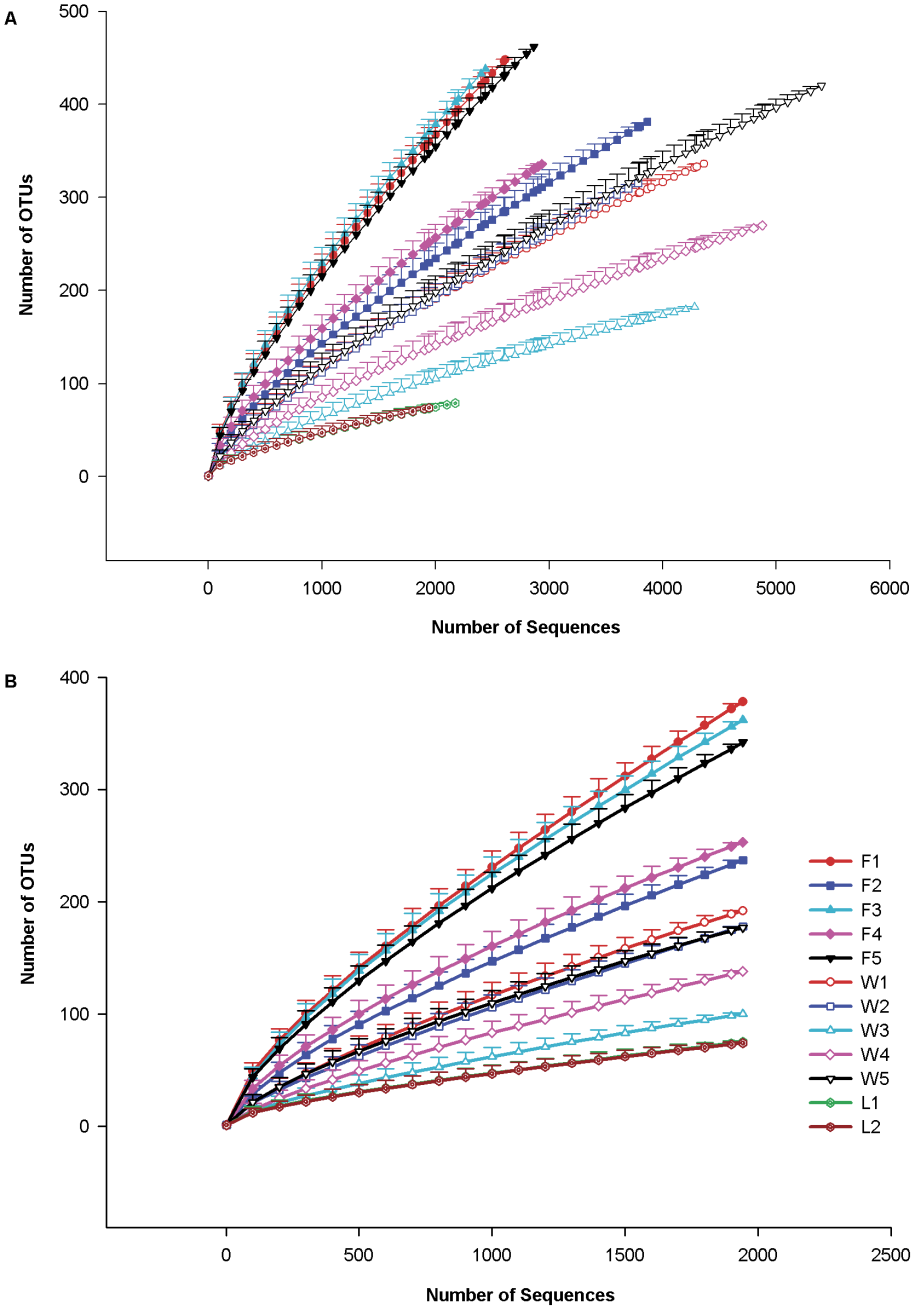
Rarefaction analysis of V8 samples from each fall, winter and lab blade showing the mean (±95% C.I.) number of OTUs (0.03 distance) as a function of sequencing depth. Note the overlap of curves for F1, F3, F5 (top); W1, W3, W5 (middle); L1, L2 (bottom). Panel A contains the analysis for samples before normalization; Panel B shows rarefaction analysis for the normalized samples.

**Figure 4:**
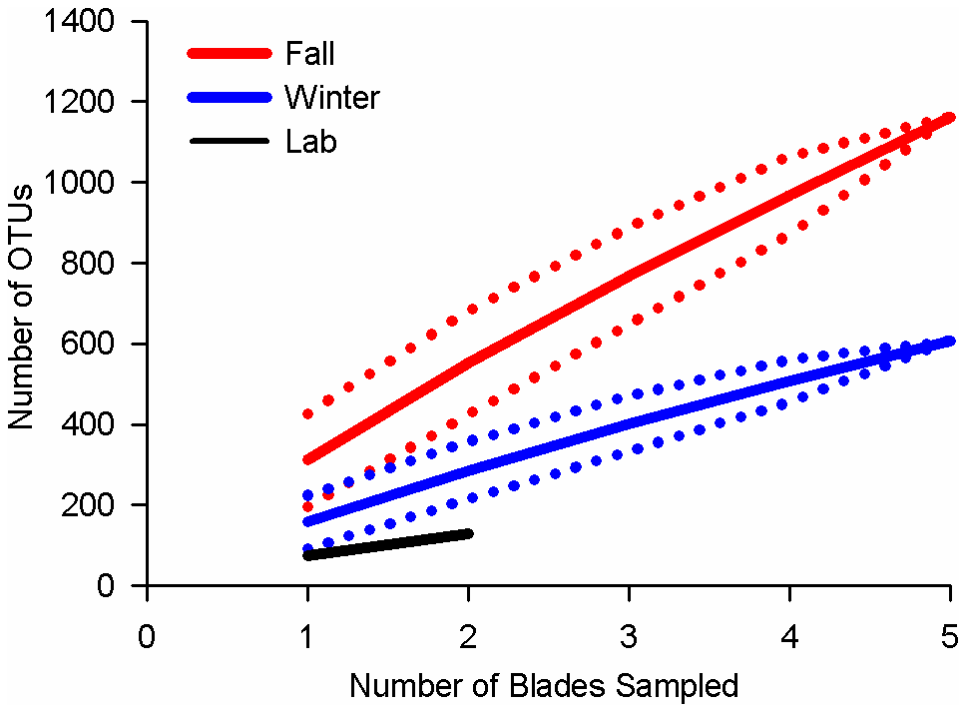
Rarefaction of sequences by number (in any order) of blades sampled in each group within the V8 library (i.e., “1” on the x-axis would not refer to either F1 or W1). Means (±95% C.I. as broken lines) are plotted.

A total of 2,526 OTUs were formed at 0.03 distance from sequences in the V8 library (Table S3 in File S1). Most of these OTUs were formed from only 1 sequence (73.8%) and only 3.6% of OTUs were comprised of ≥20 sequences. Rarefaction analysis of individual samples (Fig. 3) before and after normalization found that the sampling depth was best at describing the microbial community in the laboratory blades and some winter blades; rarefaction curves approached a plateau for L1, L2, W3 and W4. To determine how well the sampling depth described richness by group (fall vs winter vs lab), we considered rarefaction over blades within each group in mothur (Fig. 4). This analysis showed that a sample size of 5 blades was too low to describe the microbial community of *P. umbilicalis* at the fall sampling date; this sample size was more suitable for the winter time point, although additional sampling would clearly find additional OTUs (0.03 distance). The group rarefaction analysis showed that analysis of 2 blades from the clonal laboratory cultures was satisfactory (Fig. 4).

**Figure 5:**
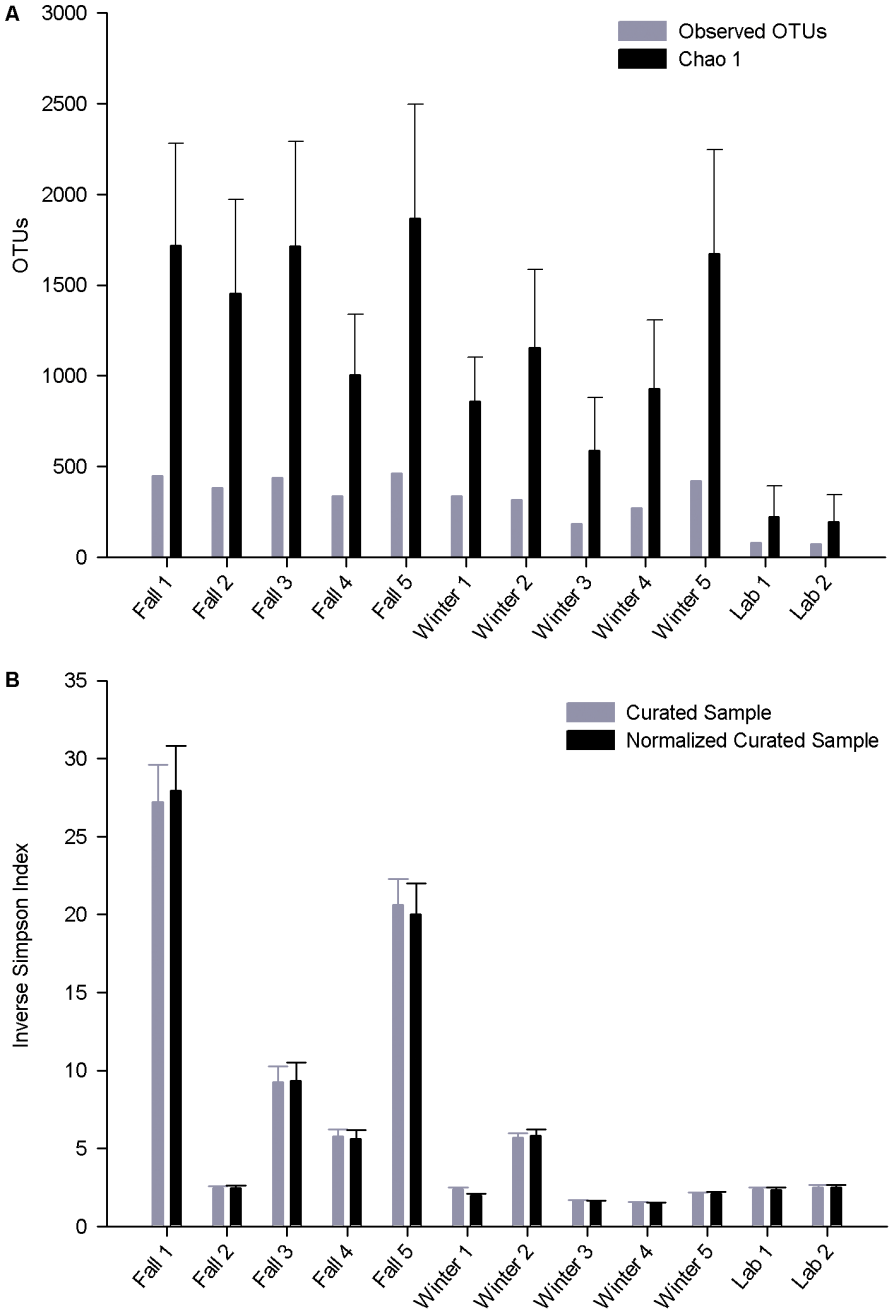
OTUs richness and diversity. Richness (A) of V8 OTUs (0.03 distance) observed and those predicted (Chao1) for samples before normalization and diversity (B) calculated for samples before and after normalization with the inverse Simpson index.

The number of OTUs observed at 0.03 (“species”), 0.05 (“genus”) and 0.10 (“family”) distance was compared to estimates of the minimum true predicted richness with the Chao1 estimator (Fig. 5, Table S4 in File S1). The number of observed OTUs at a 0.03 distance ranged between samples from 336–448 (F4, F1) for fall blades, 183–420 (W3, W5) for winter blades, and was lowest at 74–79 (L2, L1) for the lab blades. The Chao1 estimator predicted many additional OTUs: a range of 1006–1868 (F4, F5) OTUs for fall blades, 588–1672 (W3, W5) for winter blades, and 196–222 (L2, L1) for lab blades (Fig. 5, Table S4 in File S1). The relative order of observed OTU richness in individual samples was virtually identical with (L2 ≤L1≤W3 ≤W4 ≤W2 W1 = F4≤F2 ≤W5≤F3 ≤F1≤F5) and without (L2 ≤L1≤W3 ≤W4 ≤W2≤W1≤F4≤W5 ≤F2 ≤F3≤F5≤F1) considering the OTUs belonging to unclassified Bacteria in the V8 library (1,087 of the 2,526 total OTUs). Normalization of sampling depth (see Methods, File S1) reduced the total number of OTUs to 1,705 OTUs (0.03 distance) with a range of 219–369 (F2, F3) OTUs observed over individual fall samples and a Chao1 prediction of 618–1604 (F2, F1); individual winter samples ranged from 108–185 observed OTUs (W3, W5) and 440–834 predicted OTUs (W3, W5) for. Normalized samples for lab blades had 74 observed OTUs and 196 Chao1 predicted OTUs. The inverse Simpson diversity index was nearly identical for each sample before and after normalization, and it was lowest at about 1.5 for W4 and highest at 27 for F1 (Fig. 5, Table S4; W4≈ W3<W5<L1 ≈W1 ≈ L2 ≈ F2<W2 ≈ F4<F3<F5<F1). Similar trends were observed for the “genus”-level (95% similarity) and “family”-level (90% similarity) OTUs (Table S4 in File S1).

The Bacteroidetes (866 OTUs) and Proteobacteria (510 OTUs; 337 in Alphaproteobacteria and 96 in Gammaproteobacteria) were OTU-rich over all samples (Fig. 2B, Table S3). The proportion of OTUs in Bacteroidetes was high in Sphingobacteria in field blades compared to Flavobacteria, and this relationship was reversed for lab blades (Fig. 2, Table S3 in File S1). Unclassified Bacteria (Table 1) formed a mean of 37% of all fall OTUs, 41% of winter OTUs, and 18% of lab OTUs (Table S1 in File S1, Fig. 2B); most of these OTUs were represented by single sequences.

### Sample and group structure

**Figure 6:**
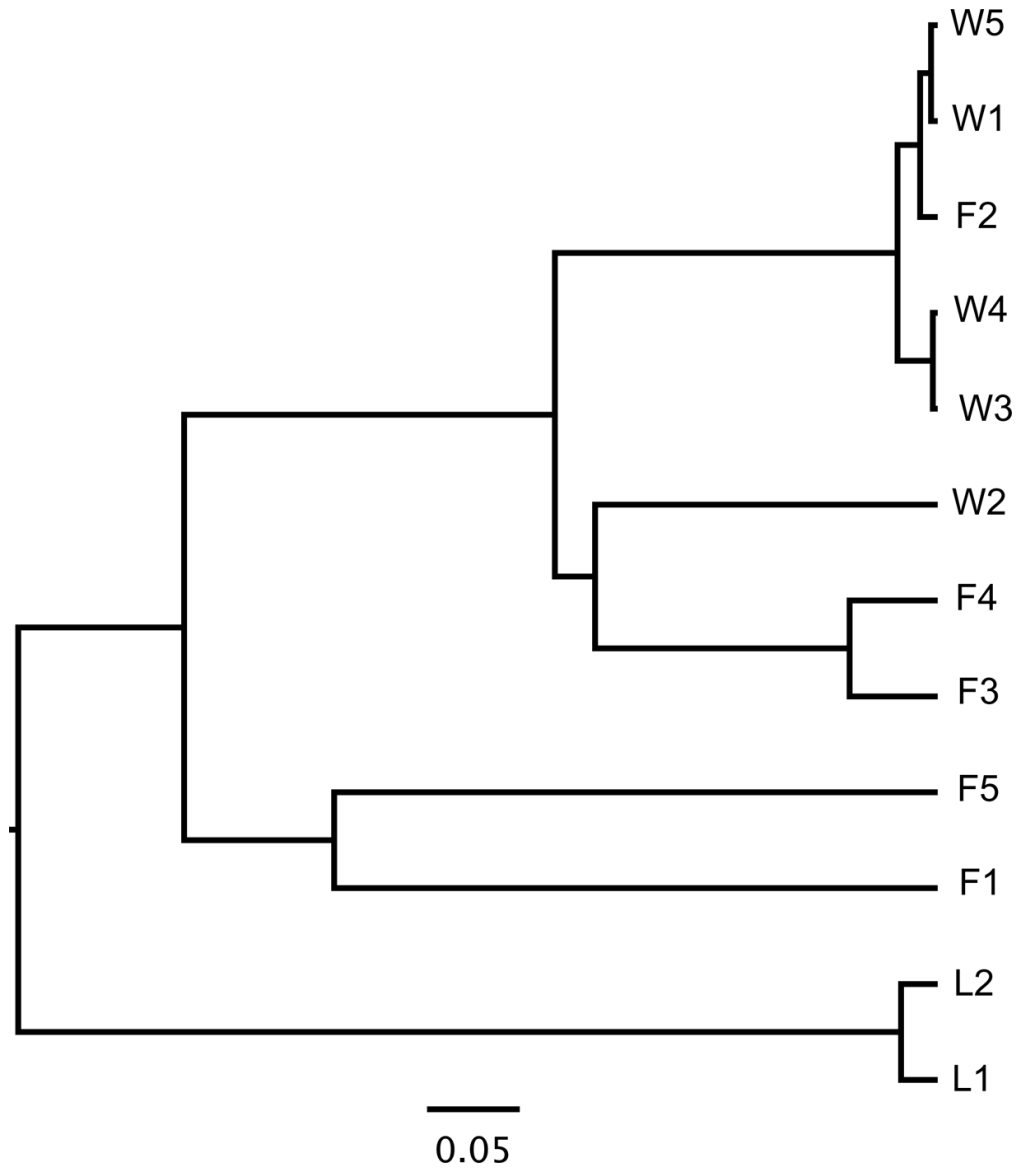
Dendrogram of the microbial community on each blade in the V8 library as clustered (thetayc distance matrix) to examine dissimilarity by group or by position along the intertidal transect for fall and winter blades.

Although subsampling reduced the number of OTUs (Table S4), the normalized community remained representative of the parent sample (cf. Fig 2A and 2C, Table S1 in File S1) and was used to test whether microbial communities on blades were dissimilar to each other and whether there was significant structure among the fall vs winter vs lab microbial communities. The dendrogram produced by clustering samples based upon the Yue-Clayton similarity coefficient (thetayc) (Fig. 6) had significant structure (branch-weighted unifrac: F-L, *p*<0.006; F-W, *p*<0.001; L-W, *p*<0.001). Microbial communities of lab blades segregated from other samples, and the position of microbial communities on fall and winter blades in the dendrogram did not relate closely to blade positions along the linear transect in the intertidal zone. A dendrogram based upon distances from the Jaccard calculator (Fig. S2) also showed significant structure (branch-weighted unifrac: F-L, *p* = 0.006; F-W, *p*<0.001; L-W, *p*<0.001). Lab communities and fall communities formed two discrete clusters, and there was more trend in the dendrogram toward relevance of the blade's transect position. The microbial communities from the three groups (fall, winter, lab) were significantly different (*p*<0.007 for group and all pair-wise tests in each analysis, Table S5 in File S1) from each other in AMOVA based on two different analyses (Jaccard, thetayc calculators, see Methods).

### The core microbiome and most abundant OTUs

We identified shared OTUs (0.03 distance) in each library (V8, V5V6) without sample normalization in order to use the greatest sequencing depth across samples to identify the number of OTUs shared among groups (i.e., sequences merged across samples within fall, winter, and lab groups). OTUs (0.03 distance) formed from V5V6 sequences for this analysis represented 4,599 OTUs, and 2,309 of these were found in Bacteria that classified to phyla and lower taxonomic levels.

**Figure 7:**
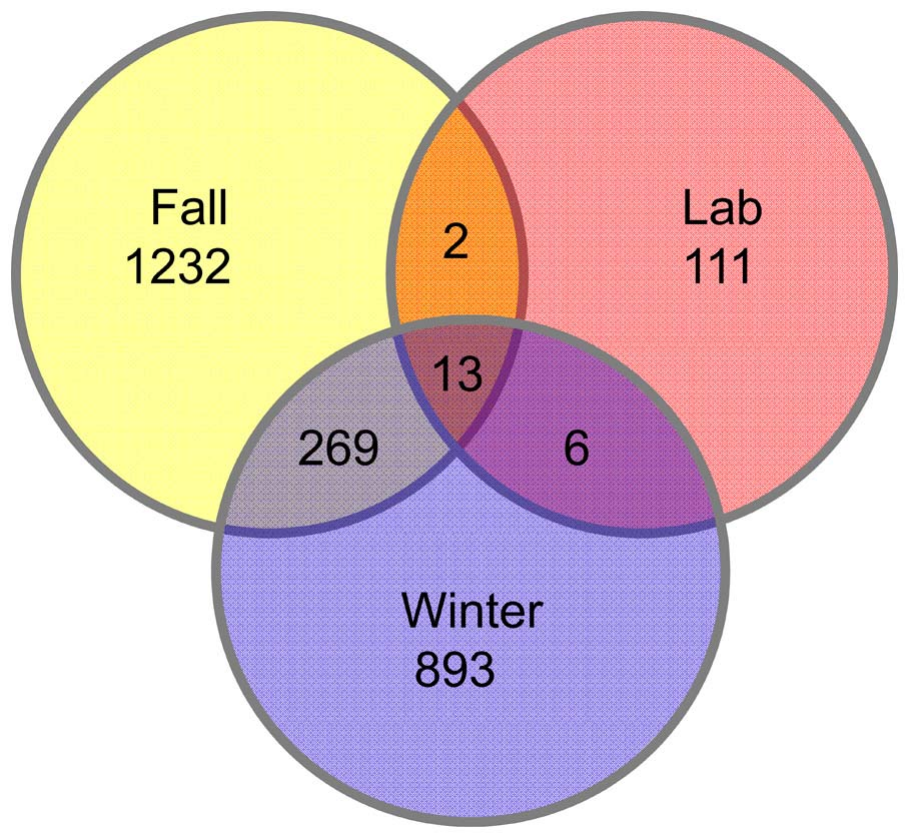
Venn diagram showing the number of OTUs (0.03 distance) that are shared between and among groups; for example, 13 bacterial OTUs are shared by at least one blade in all three groups (fall, winter, laboratory).

**Figure 8:**
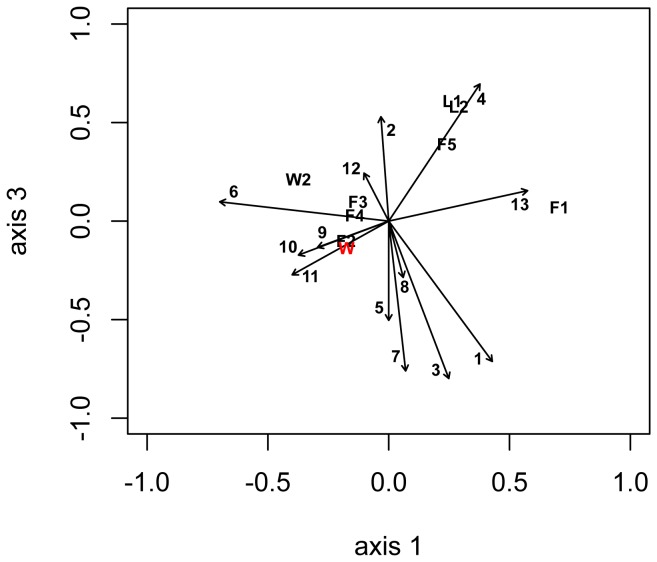
Biplot of all blade samples in a nmds ordination (here showing an axis1-axis 3 plot) with blade OTUs that belong to the core microbiome described from the V8 library. Vectors pointing toward “axis 3” are associated with winter samples; vectors pointing toward the bottom “axis 1” label are associated with fall samples, and vectors pointing away from the “axis 1” label are associated with laboratory samples. The OTU's length indicates the strength of the association, and the direction indicates the direction of the effect; the relative positions of each sample (n = 12) are plotted in the biplot. Numbers for OTUs in the figure correspond to OTU reference numbers as follows and 9 OTUs are significantly associated with an axis at *p*<0.05: 1 = OTU 1982; 2 = OTU 2387; 3 = OTU 2410; 4 = OTU 2427; 5 = OTU 2434; 6 = OTU 2521; 7 = OTU 2525; 8 = OTU 2493(NS); 9 = OTU 2408; 10 = OTU 1933(NS); 11 = OTU 2219(NS); 12 = OTU 2494(NS); 13 = OTU 2225; for taxonomic classification see Table 4; also see Table S9 for significantly associated OTUs.

The core microbiome (Fig. 7, Table 3) of *P. umbilicalis* based upon the V8 analysis found 13 OTUs in common, and 11 OTUs had an abundance of at least 1% of all seqeuences on at least one of the 12 blades, including *Granulosicoccus* (Gammaproteobacteria). Eight of the core taxa were members of the Sphingobacteria; four of these belong to the Saprospiraceae, including one found across all 12 blades at high abundance, being represented by more than 1% of all sequences from each blade (OTU 2521, see Table 3.A). As with the full community analyzed above, the majority (12 of 13) of the core OTUs were Bacteroidetes and Proteobacteria, including four OTUs belonging to Alphaproteobacteria. OTU 2494 of the Hyphomonadaceae (Alphaproteobacteria) was also present on all 12 blades, but it was uncommon on most field blades. The sampling depth (Table 1) across fall (n = 14,714 sequences) and winter (n = 22,683 sequences) was about 2× higher for winter in the curated sequences, but among these core F-W-L OTUs, six were ≥3× more abundant in winter than fall, four were evenly distributed, and two were ≥3× more abundant in fall than winter (Table 3). In each season, there was taxonomic diversity in the core microbiome (Table 3, Fig. 8, Table S9 in File S1), and although most of these OTUs were very abundant, several were not.

**Table 3:** Core and abundant OTUs found in the *Porphyra umbilicalis* microbiome identified using V8 (Taxonomic level 2 = phylum, 3 = class, 4 = order, 5 = family, 6 = genus).

**A. Core Microbiome^a^**			
**Tax level**	**Taxon**	**No. of OTUs**	**OTU code number**
2	Bacteroidetes	7	
3	Sphingobacteria	4	
5	Saprospiraceae	4	1982(F)*, 2434(E)*, 2521(W)**, 2525(E)*
3	Flavobacteria	2	
5	Flavobacteriaceae	2	2410(F)*, 2427(W)*
3	Unclassified	1	2387(W)
2	Chloroflexi	1	2493(E)*
2	Proteobacteria	5	
3	Gammaproteobacteria	1	
6	*Granulosicoccus*	1	2408(W)*
3	Alphaproteobacteria	4	
5	Hyphomonadaceae	1	2494(W)* ^L^
5	Rhodobacteraceae	1	2225(E)* ^L^
5	Unclassified	2	1933(E), 2219(W)*
**B. Lab: Most abundant OTUs^b^**			
**Tax level**	**Taxon**	**No. of OTUs**	**OTU code number**
2	Bacteroidetes	3	
3	Sphingobacteria	1	
5	Saprospiraceae	1	2521
3	Flavobacteria	2	
5	Flavobacteriaceae	2	2367, 2427
2	Planctomycetes	1	1352
2	Proteobacteria	4	
3	Gammaproteobacteria	1	
5	Alteromonadaceae	1	2182
3	Alphaproteobacteria	1	
5	Rhodobacteraceae	1	2225
5	Sphingomonadaceae	1	
6	*Sphingopyxis*	1	1938
**C. Field: Most abundant OTUs^c^**			
**Tax level**	**Taxon**	**No. of OTUs**	**OTU code number**
2	Bacteroidetes	9	
3	Sphingobacteria	9	
4	Sphingobacteriales	3	1875, 2381, 2383
5	Saprospiraceae	4	2434, 2497, 2521, 2525
6	*Lewinella*	2	2302, 2475
3	Flavobacteria	1	
5	Flavobacteriaceae	1	2410
3	Unclassified	1	2435
2	Proteobacteria	1	
3	Alphaproteobacteria	1	2514
2	Unclassified (Bacteria)	3	2489, 2500, 2524

a 13 OTUs (0.03 distance) found across fall, winter & lab groups.

* present at≥1% of all sequences on at least one blade (n = 12)

* ^L^≥1% on a lab blade

** present at ≥1% on all 12 blades

b OTUs (0.03 distance) with sequence abundance greater than 1% on both lab blades

c OTUs (0.03 distance) with sequence abundance greater than 1% on at least 3 of the 10 winter (n = 5) and fall (n = 5) blades; underlined OTUs present on at least 5 of 10 blades; OTU 2521 present on all.

Designations of F = Fall, E = Evenly distributed between fall and winter, W = Winter refer to a ≥3× skew in sequence abundance toward that group.

Fall and winter microbial communities on blades shared 269 OTUs (Fig. 7). The shared OTUs (Table S6 in File S1) belonged to *Iamia* (Actinobacteria); *Aureispir*a, *Haliscomenobacter, Lewinella,* and unclassified Sphingobacteriales including Saprospiraceae and Chitinophagaceae (Sphingobacteria); *Tenacibaculum* (Flavobacteria); *Rhodopirellula* (Planctomycetacia); *Hellea, Loktanella*, and a large proportion of unclassified Rhodobacteraceae (Alphaproteobacteria); and *Granulosicoccus* (Gammaproteobacteria).

**Figure 9:**
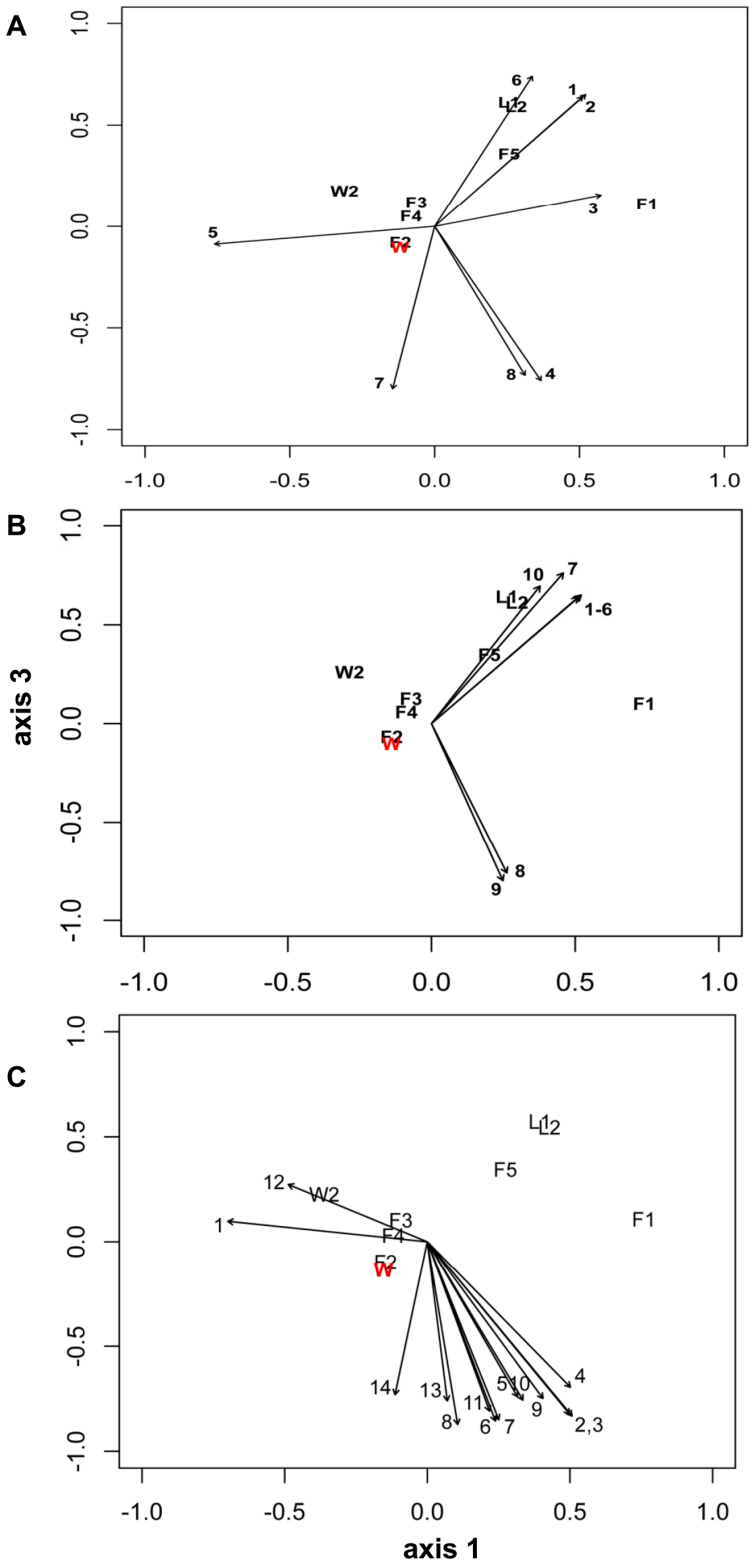
Biplots of OTUs which represent subsets of all (n = 67) of the OTUs that have significant association (*p*<0.05) with one of the nmds axes. These OTUs belong to Alphaproteobacteria (Fig. 9A), Flavobacteriaceae (Fig. 9B) and Saprospiraceae (Fig. 9C). The OTU's length indicates the strength of the association, and its direction indicates the direction of the effect; the relative positions of each sample (n = 12) are plotted in the biplot (see Table S9 and Fig. S2 for additional information on the OTUs plotted in each panel, which are decoded as follows: Fig. 9A (Alphaproteobacteria): 1 = OTU 1348, 2 = OTU 1318, 3 = OTU 2225; 4 = OTU 984; 5 = OTU 2178; 6 = OTU 1938; 7 = OTU 1763; 8 = OTU 2394; Fig. 9B (Flavobacteriaceae [Bacteroidetes]): 1 = OTU 1305, 2 = OTU 1322, 3 = OTU 1308, 4 = OTU 1328, 5 = OTU 1297, 6 = OTU 1991, 7 = OTU 2367, 8 = OTU 2071, 9 = OTU 2410; 10 = OTU 2427. Fig. 9C (Saprospiraceae [Bacteroidetes]): 1 = OTU 2521, 2 = OTU 994, 3 = OTU 1196, 4 = OTU 1150, 5 = OTU 1723, 6 = OTU 2426, 7 = OTU 1442, 8 = OTU 2302 (*Lewinella*), 9 = OTU 1001 (*Lewinella*), 10 = OTU 1558 (*Lewinella*), 11 = OTU 844, 12 = OTU 2332, 13 = OTU 2525, 14 = OTU 1674 (*Lewinella*).

Twenty-three of the most sequence-rich OTUs in the V8 library (N.B. 37 OTUs contained ≥1% of all sequences on at least one field blade) were at least 3× more abundant in fall compared to winter (Table S7 in File S1). Of the eight OTUs that had ≥1% abundance on at least one lab blade, six OTUs were more than 3× as abundant in winter, and 3 OTUs were not found among fall sequences (Table S7 in File S1). The most abundant OTUs in field samples (Table 3) included ones belonging to the Saprospiraceae (Sphingobacteria) including two of the 66 OTUs classified to *Lewinella* (Table 3, Table S3 in File S1), as well as to Flavobacteria, and Alphaproteobacteria. The most abundant OTUs on both laboratory blades (≥1%) belonged to the Bacteroidetes, Planctomycetes, and Proteobacteria including *Sphingopyxis* in Alphaproteobacteria (Table 3). Biplots (Fig. 9) of the nmds ordination of the samples and OTUs that were significantly (*p*<0.05) associated with one or more of the nmds axes demonstrated that: 1) different OTUs belonging to the Alphaproteobacteria (Fig. 9A) are associated strongly with all axes (fall, winter, lab), 2) different OTUs belonging to Flavobacteria (Fig. 9B) were significantly associated with either fall or lab blades, but not winter blades, and 3) most Saprospiraceae OTUs (Fig. 9C) significantly associated with an axis were found on fall blades but a few OTUs were significantly associated with winter blades (Fig. S3, see Table S9 in File S1 for data on the entire set of 67 significantly associated OTUs). Many other OTUs in these taxonomic groups occurred on the blades, but not in significant association with one of the nmds axes (see Fig.1, Tables S2, S3 in File S1).

*Lewinella* was always present on field blades (n = 10), but not the same OTU. Several of the most abundant OTUs in *Lewinella* were sequence-rich, and these OTUs affect distances between samples within groups in nmds plots (Fig. 9C, Table 3; OTUs 2302, 2475); however the majority of the OTUs in *Lewinella* were represented by single sequences.

The core microbiome as determined from the V5V6 sequences (Table 1) found 19 OTUs shared between fall, winter and lab samples, and 13 of these belonged to classified Bacteria within the Sphingobacteria, Flavobacteria (7 OTUs including one assigned to *Lacinutrix*), Chloroflexi, Planctomycetes (both classses including *Phycisphaera* in Phycisphaerae), and an OTU in the Alphaproteobacteria (Table 4). Two classified OTUs from Flavobacteria and Alphaproteobacteria were more than 1% abundant on both blades. OTUs present at ≥1% of total sequences of at least one field blade in the V5V6 library (Table S8 in File S1) included (Table 4) 2 Sphingobacteria, 1 Flavobacteria and 1 Chloroflexi that were identified at ≥1% abundance on at least 3 blades (n = 10). Only an OTU classified to Chloroflexi was found on most (n = 8) blades at ≥1% abundance.

**Table 4:** Core and abundant OTUs found in the *Porphyra umbilicalis* microbiome identified using V5V6 (Taxonomic level 2 = phylum, 3 = class, 4 = order, 5 = family, 6 = genus).

**A. Core Microbiome^a^**			
**Tax level**	**Taxon**	**No. of OTUs**	**OTU code number**
2	Bacteroidetes	9	
3	Sphingobacteria	2	
4	Sphingobacteriales	2	4509(W)*, 4580(W)*
3	Flavobacteria	6	
5	Flavobacteriaceae	6	3834(E), 3913(E), 3944(F), 3969(E), 4507(F)*, 4510(W)* ^L^
2	Chloroflexi	1	4599(W)*
2	Planctomycetes	2	
3	Phycisphaerae	1	
5	Phycisphaeraceae	1	
6	*Phycisphaera*	1	3875(W)
3	Planctomycetacia	1	
5	Planctomycetaceae	1	3994(F)*
2	Proteobacteria	1	
3	Alphaproteobacteria	1	
5	Rhodobacteraceae	1	4349(W)* ^L^
2	Unclassified(Bacteria)	6	2742(E), 4252(F)*, 4368(E), 4392(F)*, 4453(W),4491(E)*
**B. Lab: Most abundant OTUs^b^**			
**Tax level**	**Taxon**	**No. of OTUs**	**OTU code number**
2	Bacteroidetes	1	
3	Flavobacteria	1	
5	Flavobacteriaceae	1	4510
2	Proteobacteria	1	
3	Alphaproteobacteria	1	
5	Rhodobacteraceae	1	4349
2	Unclassified (Bacteria)	1	2443
**C. Field: Most abundant OTUs^c^**			
**Tax level**	**Taxon**	**No. of OTUs**	**OTU code number**
2	Bacteroidetes	3	
3	Sphingobacteria	2	
4	Sphingobacteriales	2	4509, 4580
3	Flavobacteria	1	
5	Flavobacteriaceae	1	4507
2	Chloroflexi	1	4599
2	Unclassified (Bacteria)	2	4392, 4491

a 19 OTUs (0.03 distance) shared between Fall, Winter & Lab Groups

* present at ≥1% of all sequences on at least one blade (n = 12)

* ^L^ ≥1% on a lab blade

b OTUs (0.03 distance) with sequence abundance greater than 1% on both lab blades

c OTUs (0.03 distance) with sequence abundance greater than 1% on at least 3 of the 10 winter (n = 5) and fall (n = 5) blades

Designations of F = Fall, E = Evenly distributed between fall and winter, W = Winter refer to a ≥3× skew in sequence abundance toward that group.

### Environmental conditions

Environmental conditions were different preceding the two different collection times on the Maine shore. October air (7–16°C) and water (11–13°C) temperatures were higher, and the photoperiod was longer (12 L:12 D) in contrast to December when air (−5–10°C) and water (7–9.5°C) temperatures were lower and the photoperiod was shorter (9 L:15 D)

## Discussion

Pyrosequencing of microbial communities associated with *Porphyra umbilicalis* identified 8 phyla containing 76 genera, including 48 genera that have been isolated and formally described. Bacteria belonging to the Bacteroidetes (especially Sphingobacteria) were most abundant and also comprised the most species-level OTUs; however, the Proteobacteria, especially Alphaproteobacteria, were also abundant and OTU-rich. Clonal laboratory blades of “P.um.1”, the *P. umbilicalis* isolate that is part of a whole genome sequencing project, had much reduced microbial richness and diversity compared to field blades, and most bacteria remaining on blades were Bacteroidetes belonging to the Flavobacteria. The two days when we sampled blades from the Maine shore were in mid-fall and winter, and several statistical comparisons found that the microbial communities were different from each other on these two days and also that each was different from the microbial community on lab blades. Fall blades had the highest observed and predicted (Chao1) richness and the highest diversity (inverse Simpson Index). Only two OTUs were found on all blades in this study, and only an OTU belonging to the Saprospiraceae (Bacteroidetes) was found abundantly on all blades. Although the genus *Lewinella* was found on all field blades, none of its 66 OTUs was universally present on these blades. This pyrosequencing study increases our understanding of the microbial communities of the red algae and makes it possible to compare the bacteria associated with marine red and green algae, which have different cell wall compositions but are two of the three known groups of algae formed in primary endosymbiosis.

### Algal microbial communities and the core microbiome

The microbial communities of two red macrophytes from temperate latitudes, *Delisea pulchra* [22] and *Gracilaria vermiculophylla* [25] were studied with 16 S rDNA clone libraries, and over 5,000 nearly full length 16 S rDNA seqeuences recently described [24] the microbial community of *Ulva australis* (Chlorophyta). These offer among the best comparisons available to our results on *P. umbilicalis*. The microbial community of *G. vermiculophylla* is of special interest because it has agar-rich cell walls, sufated galactans that are structurally similar to porphyran in the *Porphyra* cell wall [63]. Lachnit et al. [25] found that *Gracilaria* was richest in Bacteroidetes followed by Proteobacteria, as found for *P. umbilicalis* in the present study. They found temporally consistent differences in the composition of the microbial community at the level of phylum between *Gracilaria*, the green alga *Ulva* and the brown alga *Fucus vesiculosus* from individuals collected within 20 m of each other. Longford et al. 's similar study [22] near Sydney, Australia, found representatives of 7 phyla on *Delisea pulchara* (Rhodophyta); Bacteroidetes and Proteobacteria were also prominent on the red alga *Delisea* in that study. More recently, Burke et al. [24] examined the microbial community of *U. australis* with over 5,000 nearly full-length 16 S rDNA sequences, finding that 74% of all sequences classified to Alphaproteobacteria (Proteobacteria) and 13% to Bacteroidetes. Given that only 6 OTUs (a *Lewinella*, 4 unclassified Rhodobacteriaceae and *Erythrobacter*) were found in common across the 6 samples of *Ulva* blades in their study, they proposed [24] that *Ulva*'s obligately symbiotic microbes must operate as a lottery that in functionally-equivalent, but OTU-distinctive, subsets can supply the host with its microbial requirements (e.g., vitamins, and plant growth regulators such as the morphogen thallusin). This concept was supported by their recent metagenomic study [27], which showed a highly shared functional genome across different microbial taxa occupying the surfaces of blades, despite the low similarity in the composition of species-level OTUs on individual blades [24]. Our study suggests something similar for *Porphyra* blades, because even using the higher sequencing depth of pyrosequencing, only 1 OTU from the Saprospiraceae was found in common over all 12 blades in our study. However, the natural microbial community of the temperate Northern Hemisphere *P. umbilicalis* has some striking similarity to that reported from the temperate Southern Hemisphere *U. australis* [24] (cf. our Fig 1 and Tables S1–S3 in File S1 [24]). At the level of genus, this includes the Saprospiraceae *Lewinella* and *Haliscomenobacter*, unclassified Flavobacteriaceae, unclassified Planctomycetaceae (as well as *Rhodopirellula* for *P. umbilicalis*), and the Proteobacteria *Krokinobacter, Loktanella, Maribacter, Roseovarius*, unclassified Roseobacteriaceae, and *Winogradskyella*. The widespread level of horizontal gene transfer among taxa within and among microbial taxa [64]–[65] supports the concept of a lottery of species, but there still appear to be some particular macrophyte-associated taxa that deserve special attention in understanding microbial-macroalgal interactions.

### Variability in recovery of microbial taxa in the V5V6 library compared to V8 library

There was little variation in the taxa found to be present between the two libraries, especially when taking into account the nearly 2× higher coverage of the V5V6 library. However, Chloroflexi were abundant in the V5V6 library compared to being a small component of the microbial community in the V8 library. Both Deinococcus-Thermus and TM7 were recognized at trace levels in sequences within the V8 and V5V6 libraries, respectively; additional sampling depth in either might reveal both phyla. It is well-known that the 16 S rDNA evolves at different rates along its length [66], which contributes to some variability in description of the microbial community when different variable regions of the 16 S gene are used. As reported in the Results, the primers we used to assay the V5V6 regions recognized more Flavobacteria compared to V8 in our study (Fig. 1), and V8 recognized more Sphingobacteria than V5V6. In terms of the core microbiomes, both primer sets recovered a Bacteroidetes-rich core, but the V5V6 library contained additional Flavobacteria compared to the V8 library. The V5V6 library also contained sequences classified to both Planctomycetes' classes including Phycisphaerea. The core microbiome found from the V8 library contained more Proteobacteria. Thus, it can be useful to use more than one set of variable regions in pyrosequencing to understand the microbial community, but shorter tags (e.g., V6 vs V5–V7) may improve analysis, especially as the number of described microbial taxa increases. Our primer selection was based on being able to classify with high confidence to a genus level using the RDP Classifier (i.e., a minimum length of 200 bases, [67]); however, the two long conserved regions in our V5V7 tag may have contributed to formation of complex chimeras that made chimera recognition and removal difficult, resulting in an unusually high proportion of unclassified Bacteria in some samples. A number of factors contribute to chimera formation (e.g., see [57]).

### The P.um.1 blade community

Most of the bacteria remaining associated with the laboratory blades in terms of sequence abundance were unidentified Flavobacteriaceae although Proteobacteria and Planctomycetes remaining on/in blades had greater OTU richness. Many taxa present on field blades were eliminated from lab blades by penicillin and streptomycin but these antibiotics are not effective against many of the remaining taxa, including the Planctomycetes [68], which increased in abundance on lab blades, and several Proteobacteria (e.g. *Granulosicoccus*). Nearly all of the Sphingobacteria were lost due to antibiotic treatment of blades but the core OTU in the Saprospiraceae (OTU 2521) remains. Because the laboratory blades continue to grow and reproduce normally, although grown in medium with vitamins (McBride-West formulation of Provasoli's Enriched Seawater [48]), this greatly reduced microbial community offers a good community for metagenomic studies to elucidate required functionality for the host blade. It is notable that a significant number of OTUs that are abundant on lab blades are represented more (or exclusively) with winter blades if they occur in either the fall or winter groups; P.um.1 was isolated from the field in early April.

### Cell wall digesting bacteria and seasonal trends

The capacity to digest the polysaccharides of algal cell walls is especially associated with Bacteroidetes, Proteobacteria and Planctomycetes [65], [69]. Digestion of brown algal cell walls is also carried out by the Verrucomicrobia [70], which were found to be seasonally prominent on the brown alga *Fucus* in addition to its abundant Bacteroidetes, Proteobacteria (Alphaproteobacteria, Gammaproteobacteria) and Planctomycetes (e.g., [25]) The Alginate Utilization System, consisting of two operons, has spread through at least two independent horizontal gene transfer events from Flavobacteria to Alphaproteobacteria and Gammaproteobacteria, as elucidated from genomic studies of *Zobellia galactanivorans* [65]. Many Bacteroidetes on *P. umbilicalis* (see Results) including *Zobellia, Aquimarina,* and some *Lewinella* also obtain carbon from digestion of agar and porphyran in red algal cell walls [63], [71], [72], and it would not be surprising if the genes encoding agarases and porphyranases had been transferred from Bacteroidetes to Proteobacteria (and some other taxa), as demonstrated for the Alginate Utilization System. Indeed, two cases are already known of horizontal gene transfer of the alginolytic operon [65] and porphyranase [73] from *Zobellia* to gut bacteria of the Japanese, who traditionally consume large quantities of brown and red macroalgae for food. Thus, many of the bacteria that live on the *P. umbilicalis* blade likely benefit from this association by obtaining their carbon from digestion of the cell wall.

Our analysis showed that the microbial communities on *P. umbilicalis* were different between fall and winter sampling days. The richness of the microbial communities on the fall blades may be related to benign environmental conditions compared to winter, and also to this time representing an early stage in recovery of blades from the environmental stresses they experience in summer. In summer (July, August) blades experience many stresses (e.g., low nutrients in stratified seawater, hot and dry intertidal periods); neutral spores that are made are not viable and blades frequently have higher epiphyte loads before dying back to smaller blades [37]. The blades we collected in the field were healthy and reproductive but likely represent a transitional state between summer and winter communities.

The observed variability in the number of OTUs and species diversity within blades collected within the same season may be due in part to biotic factors that differentially affect blades. For example, there is differential grazing pressure from amphipods and isopods (Crustacea) as well as small *Littorina littorea* snails. Further, migrating ducks and geese pass through this area in the fall, and they often float above the intertidal zone at high tide. Blade position on the transect showed some trend to be important in community similarity between blades based upon OTU presence or absence (Jaccard analysis) but little such trend in the analysis (thetayc) that considered abundance as well as membership of OTUs in calculating dissimilarity of blade communities. A larger number of blades is required to examine the spatial relationships of microbial communities, especially in fall, based upon the rarefaction analysis done over blades within each group.

Macroalgae live in a very dynamic environment that is affected by diverse biotic and abiotic factors; all of these will contribute to shaping the bacterial community associated with the blade and producing diversity in the microbial communities of different blades within a species. Despite these diversifying factors, some patterns in the assemblages of bacteria found on macroalgae that are attributable to the different cell wall materials found in host walls are evident, and also some conservation of the most abundant phyla of microbes on the host (e.g., Bacteroidetes, Proteobacteria).

### Did macroalgae evolve as bacterial farms?

Given the greater understanding of the microbiome of macroalgae that is emerging from this study and others (e.g., [22], [24], [25]), there is a striking overlap between bacteria known to have cell-wall digesting capacity and those known to have the ability to maintain the normal morphology of macroalgae, including the large sheet-like blades of *Ulva* (Chlorophyta) and *Porphyra* (Rhodophyta). Without particular bacteria, macroalgae literally fall apart, into simple filaments and single cell suspensions [12], [18], [21]. There is probably more than one genetic pathway by which bacteria support algal morphogenesis [cf. 16, 74], but the thallusin strategy was strongly localized to a subset of Bacteroidetes in a clade that includes the Flavobacteria *Zobellia* and *Cytophaga* [18]. We propose a model in which this synergy---microbial cell wall digestion to acquire carbon and algal cell wall production to maintain bacterial symbionts that also provided vitamins, inorganic nutrients and plant growth regulators---could have provided strong natural selection to produce large, stable habitats for these microbes---macroalgae---from single-celled ancestors. These single-celled ancestors could literally have become entrapped in the bacterial stimulated farm (cell walls).

The Bangiophyceae that *Porphyra* belongs to are an ancient group in a phylum in which classes closer to the ancestral rhodophyte are composed nearly entirely of taxa with simple unicellular and filamentous morphologies (reviewed by [75]). Complex and much larger morphologies are found in the Bangiophyceae and its sister class, the Florideophyceae (e.g., *Chondrus, Gracilaria*). It is important to point out that there appear to be “cheaters” among the bacteria in terms of our model; not all strains of *Cytophaga* (Bacteroidetes) supported normal morphogenetic development in *Ulva linza* [17], for example. Microbial morphogen production appears expensive, because it is not sustained in the absence of the host [12], and microbial “cheaters” among the morphogen-producers in the presence of a host macroalga can be explained by the Black Queen hypothesis [76]. This hypothesis is based upon the ability of microbes to lose part of their genome when that function is being provided by other microbes that are closely enough associated to provide that function (e.g., thallusin in the case of microbes, various microbially supplied vitamins in the case of algae). As the ancient symbioses between some bacteria and algae developed, “cheating” by the algae also appears to have evolved in a highly regulated way (e.g., requirements for bacterial supplied vitamins [9]). Continued exploration of the under-studied microbiome of macroalgae is needed to elucidate the genomic pathways by which these elegant dependencies evolved and are maintained, and such studies are sure to result in microbial information that will have commercial applications [1], [63], [77].

## Supporting Information

Figure S1 **Richness of V8 OTUs (0.03 distance) observed and those predicted (Chao1) for normalized samples.**
(TIF)

Figure S2 **Dendrogram of the microbial community on each blade in the V8 library as clustered (Jaccard coefficient) to examine dissimilarity by group or by position along the intertidal transect for fall and winter blades.**
(TIF)

Figure S3 **Biplot of all 67 OTUs significantly associated with an nmds axis in a plot of V8 blade samples. See Table S9 for additional information on each OTU.**
(TIF)

File S1 **Combined file for the supporting information tables.**
(DOCX)
