# Supplementary figures and images for: Diversity and Abundance of the Bacterial Community of the Red Macroalga Porphyra umbilicalis: Did Bacterial Farmers Produce Macroalgae?
Source: PLoS One. 2013 Mar 20;8(3):e58269. doi: 10.1371/journal.pone.0058269 (PMC3603978; doi:10.1371/journal.pone.0058269)

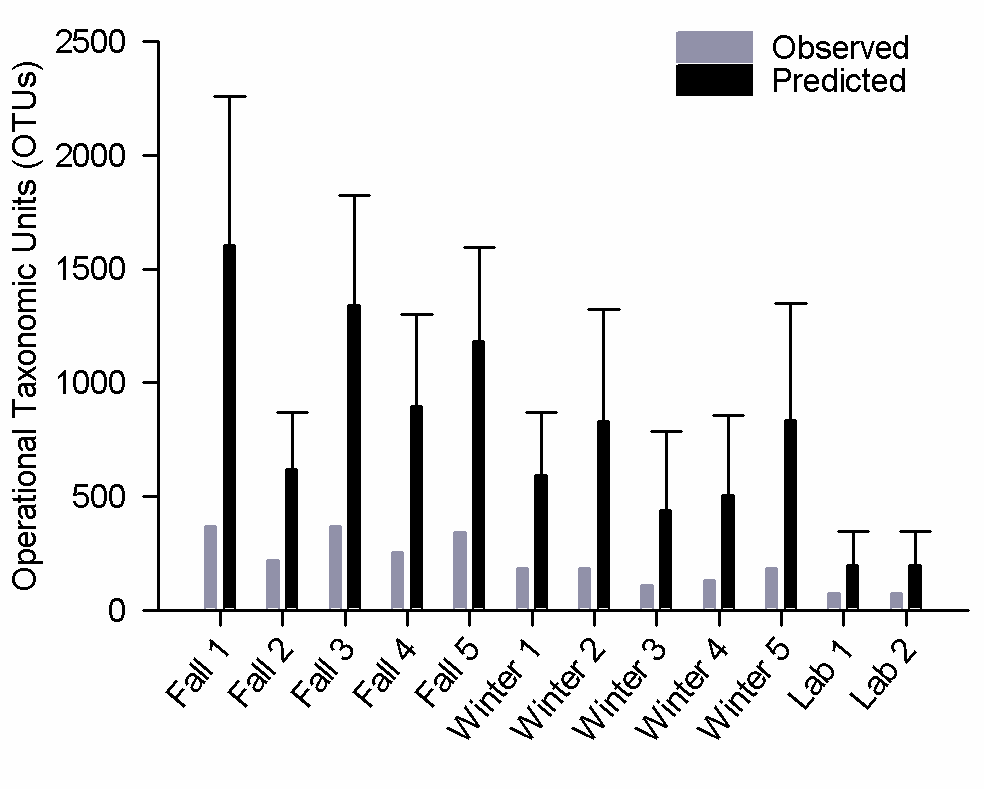

Supplement: Figure S1 — Richness of V8 OTUs (0.03 distance) observed and those predicted (Chao1) for normalized samples. (TIF) [file pone.0058269.s001.tif]

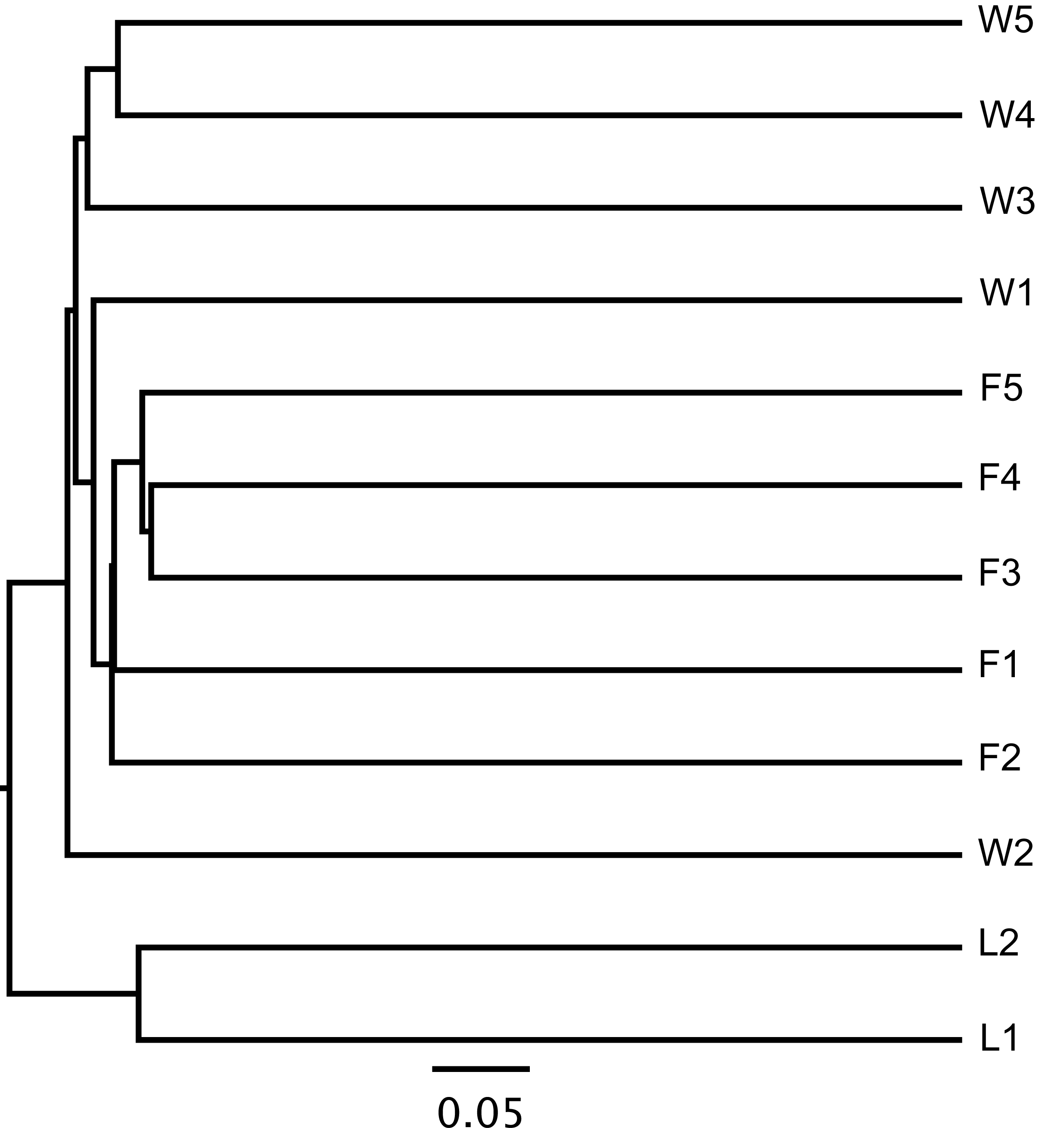

Supplement: Figure S2 — Dendrogram of the microbial community on each blade in the V8 library as clustered (Jaccard coefficient) to examine dissimilarity by group or by position along the intertidal transect for fall and winter blades. (TIF) [file pone.0058269.s002.tif]

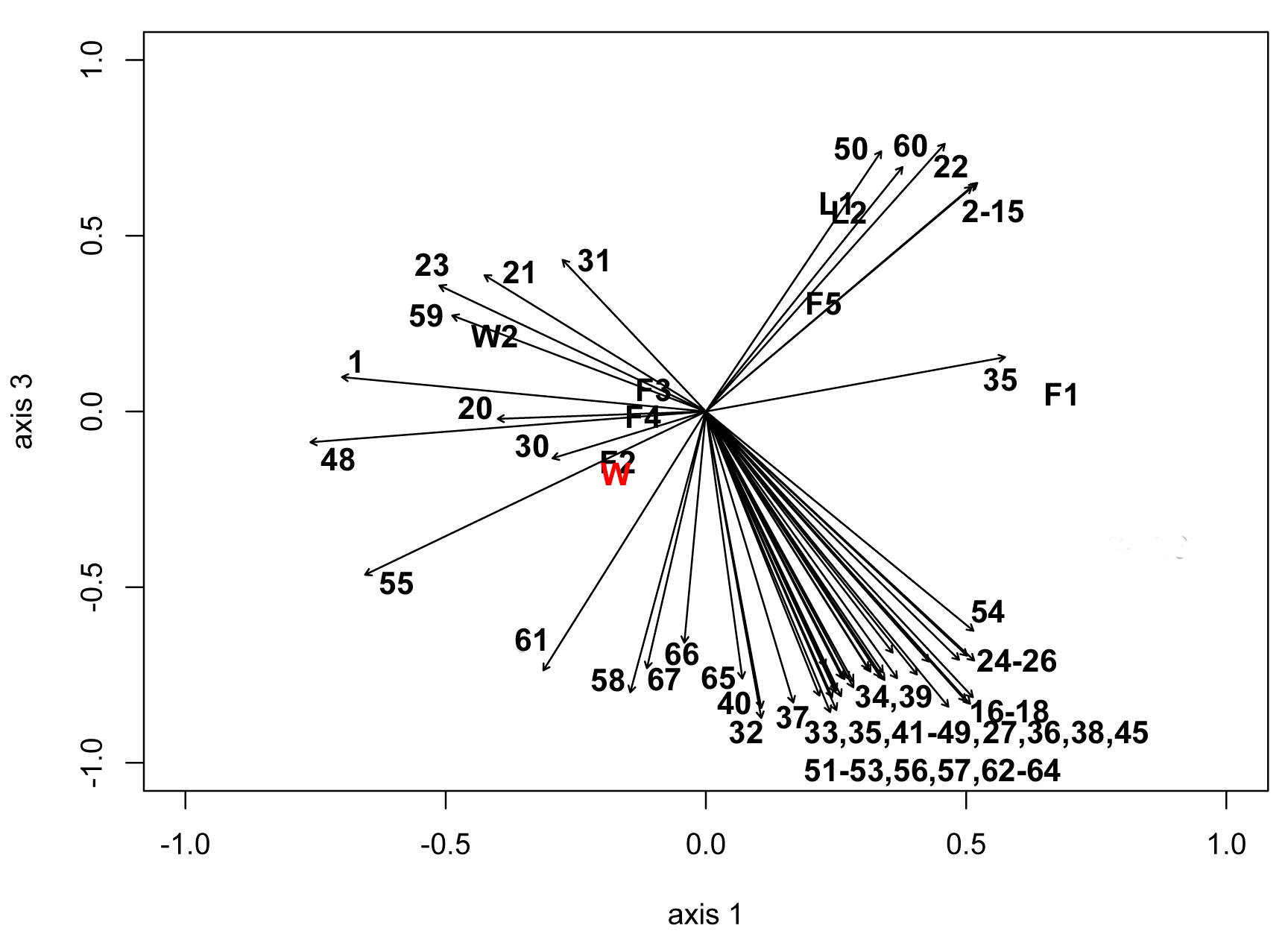

Supplement: Figure S3 — Biplot of all 67 OTUs significantly associated with an nmds axis in a plot of V8 blade samples. See Table S9 for additional information on each OTU. (TIF) [file pone.0058269.s003.tif]
