# Supplementary material for: Diversity and Abundance of the Bacterial Community of the Red Macroalga Porphyra umbilicalis: Did Bacterial Farmers Produce Macroalgae?
Source: PLoS One. 2013 Mar 20;8(3):e58269. doi: 10.1371/journal.pone.0058269 (PMC3603978; doi:10.1371/journal.pone.0058269)
Supplement: File S1 — Combined file for the supporting information tables. (DOCX) [file pone.0058269.s004.docx]

Table S1. Data (% abundance) used to make pie charts in Figure 2 using means for phyla and classes. Means ± standard deviations are given for fall samples [n=5], winter samples [n=5], and laboratory samples [n=2] for V8 sequences before normalization (A), V8 OTUs formed at 0.03 distance (B), and the normalized subsample of V8.

| **A. Before Normalization** |  |  |  |  |  |  | |
| --- | --- | --- | --- | --- | --- | --- | --- |
| **Taxon** | **Mean Fall** | **SD Fall** | **Mean Lab** | **SD Lab** | **Mean Winter** | **SD Winter** | |
| Actinobacteria | 1.49% | 1.03% | 0.00% | 0.00% | 0.14% | 0.133% | |
| Sphingobacteria | 60.41% | 9.29% | 1.30% | 0.38% | 73.63% | 12.408% | |
| Flavobacteria | 4.12% | 3.84% | 79.23% | 4.78% | 0.63% | 0.515% | |
| unclassified Bacteroidetes | 6.17% | 3.78% | 0.32% | 0.06% | 4.83% | 1.490% | |
| unclassified Chloroflexi | 1.37% | 2.07% | 0.05% | 0.07% | 1.07% | 1.725% | |
| Deinococci | 0.01% | 0.01% | 0.00% | 0.00% | 0.00% | 0.000% | |
| Planctomycetacia | 1.10% | 1.37% | 4.06% | 1.22% | 0.03% | 0.045% | |
| Phycisphaerae | 0.00% | 0.00% | 0.00% | 0.00% | 0.01% | 0.033% | |
| unclassified Planctomycetes | 0.01% | 0.02% | 0.00% | 0.00% | 0.00% | 0.000% | |
| Alphaproteobacteria | 7.12% | 4.49% | 7.96% | 1.36% | 10.89% | 16.653% | |
| Betaproteobacteria | 0.00% | 0.00% | 0.00% | 0.00% | 0.00% | 0.010% | |
| Deltaproteobacteria | 0.04% | 0.05% | 0.00% | 0.00% | 0.00% | 0.000% | |
| Gammaproteobacteria | 1.52% | 0.38% | 6.14% | 1.99% | 1.94% | 2.047% | |
| unclassified Proteobacteria | 0.34% | 0.05% | 0.07% | 0.10% | 0.26% | 0.137% | |
| Bacilli | 0.00% | 0.00% | 0.00% | 0.00% | 0.00% | 0.009% | |
| unclassified Bacteria | 16.30% | 6.86% | 0.89% | 0.28% | 6.56% | 2.059% | |
| **B. OTU** |  |  |  |  |  |  |  |
| **Taxon** | **Mean Fall** | **SD Fall** | **Mean Lab** | **SD Lab** | **Mean Winter** | **SD Winter** |  |
| Actinobacteria | 0.91% | 0.87% | 0.00% | 0.00% | 0.50% | 0.83% |  |
| Sphingobacteria | 31.37% | 3.27% | 3.29% | 1.08% | 21.03% | 3.52% |  |
| Flavobacteria | 2.54% | 1.32% | 36.68% | 3.54% | 2.47% | 1.33% |  |
| unclassified Bacteroidetes | 8.06% | 2.09% | 3.25% | 0.77% | 9.15% | 3.14% |  |
| unclassified Chloroflexi | 0.90% | 0.44% | 0.63% | 0.90% | 1.06% | 0.68% |  |
| Planctomycetacia | 0.87% | 0.78% | 4.56% | 0.71% | 0.32% | 0.49% |  |
| Phycisphaerae | 0.00% | 0.00% | 0.00% | 0.00% | 0.19% | 0.43% |  |
| unclassified Planctomycetes | 0.04% | 0.10% | 0.00% | 0.00% | 0.00% | 0.00% |  |
| Alphaproteobacteria | 13.78% | 6.91% | 22.03% | 8.22% | 15.53% | 13.10% |  |
| Betaproteobacteria | 0.00% | 0.00% | 0.00% | 0.00% | 0.11% | 0.25% |  |
| Deltaproteobacteria | 0.24% | 0.27% | 0.00% | 0.00% | 0.00% | 0.00% |  |
| Gammaproteobacteria | 2.78% | 1.29% | 9.84% | 1.38% | 5.53% | 3.94% |  |
| unclassified Proteobacteria | 1.99% | 0.59% | 1.27% | 1.79% | 2.71% | 1.31% |  |
| Bacilli | 0.00% | 0.00% | 0.00% | 0.00% | 0.07% | 0.17% |  |
| unclassified Bacteria | 36.53% | 3.59% | 18.45% | 6.40% | 41.34% | 7.48% |  |
| **C. Normalized** |  |  |  |  |  |  | |
| **Taxon** | **Mean Fall** | **SD Fall** | **Mean Lab** | **SD Lab** | **Mean Winter** | **SD Winter** | |
| Actinobacteria | 3.94% | 4.05% | 0.00% | 0.00% | 0.13% | 0.21% | |
| Sphingobacteria | 59.11% | 8.56% | 1.31% | 0.33% | 73.80% | 12.83% | |
| Flavobacteria | 4.22% | 3.68% | 79.18% | 4.84% | 0.57% | 0.50% | |
| unclassified Bacteroidetes | 7.67% | 4.33% | 0.39% | 0.18% | 3.37% | 0.32% | |
| unclassified Chloroflexi | 1.36% | 1.96% | 0.05% | 0.07% | 0.97% | 1.58% | |
| Planctomycetacia | 1.06% | 1.37% | 3.96% | 1.09% | 0.02% | 0.03% | |
| Phycisphaerae | 0.00% | 0.00% | 0.00% | 0.00% | 0.02% | 0.05% | |
| unclassified Planctomycetes | 0.01% | 0.02% | 0.00% | 0.00% | 0.00% | 0.00% | |
| Alphaproteobacteria | 6.75% | 4.27% | 8.13% | 1.53% | 10.81% | 16.43% | |
| Deltaproteobacteria | 0.06% | 0.08% | 0.00% | 0.00% | 0.00% | 0.00% | |
| Gammaproteobacteria | 1.62% | 0.49% | 6.10% | 2.07% | 2.04% | 2.18% | |
| unclassified Proteobacteria | 0.45% | 0.16% | 0.08% | 0.11% | 0.24% | 0.18% | |
| Bacilli | 0.00% | 0.00% | 0.00% | 0.00% | 0.01% | 0.02% | |
| unclassified Bacteria | 13.74% | 4.87% | 0.80% | 0.18% | 8.03% | 2.77% | |

Table S2. Taxonomic summary of V8 sequence abundance (mean +/- SD) from classification of sequences from OTUs formed

at 0.03 distance for fall (n=5), winter (n=5) and laboratory (n=2) samples.

| **Tax level** | **Taxon** | **Total** | **Fall Mean** | **Fall SD** | **Lab Mean** | **Lab SD** | **Winter Mean** | **Winter SD** |
| --- | --- | --- | --- | --- | --- | --- | --- | --- |
| **0** | Root 41514 seqs |  |  |  |  |  |  |  |
| **1** | Bacteria | 100.00% | 100.00% | 0.00% | 100.00% | 0.00% | 100.000% | 0.000% |
| **2** | Actinobacteria | 0.56% | 1.49% | 1.03% | 0.00% | 0.00% | 0.138% | 0.133% |
| **3** | Actinobacteria | 0.56% | 1.49% | 1.03% | 0.00% | 0.00% | 0.138% | 0.133% |
| **4** | Acidimicrobiales | 0.07% | 0.18% | 0.12% | 0.00% | 0.00% | 0.029% | 0.039% |
| **5** | Acidimicrobiaceae | 0.02% | 0.03% | 0.04% | 0.00% | 0.00% | 0.017% | 0.023% |
| **6** | *Ilumatobacter* | 0.02% | 0.02% | 0.03% | 0.00% | 0.00% | 0.017% | 0.023% |
| **6** | unclassified | 0.00% | 0.01% | 0.02% | 0.00% | 0.00% | 0.000% | 0.000% |
| **5** | Iamiaceae | 0.04% | 0.12% | 0.12% | 0.00% | 0.00% | 0.004% | 0.008% |
| **6** | *Iamia* | 0.04% | 0.12% | 0.12% | 0.00% | 0.00% | 0.004% | 0.008% |
| **5** | unclassified | 0.02% | 0.04% | 0.04% | 0.00% | 0.00% | 0.008% | 0.011% |
| **6** | unclassified | 0.02% | 0.04% | 0.04% | 0.00% | 0.00% | 0.008% | 0.011% |
| **4** | unclassified | 0.48% | 1.31% | 0.92% | 0.00% | 0.00% | 0.109% | 0.102% |
| **5** | unclassified | 0.48% | 1.31% | 0.92% | 0.00% | 0.00% | 0.109% | 0.102% |
| **6** | unclassified | 0.48% | 1.31% | 0.92% | 0.00% | 0.00% | 0.109% | 0.102% |
| **2** | Bacteroidetes | 77.10% | 70.70% | 7.26% | 80.84% | 4.46% | 79.092% | 13.728% |
| **3** | Sphingobacteria | 62.61% | 60.41% | 9.29% | 1.30% | 0.38% | 73.632% | 12.408% |
| **4** | Sphingobacteriales | 62.61% | 60.41% | 9.29% | 1.30% | 0.38% | 73.632% | 12.408% |
| **5** | Saprospiraceae | 54.94% | 45.12% | 15.31% | 1.22% | 0.42% | 69.156% | 11.915% |
| **6** | *Aureispira* | 0.09% | 0.16% | 0.11% | 0.00% | 0.00% | 0.068% | 0.072% |
| **6** | *Haliscomenobacter* | 0.10% | 0.23% | 0.15% | 0.00% | 0.00% | 0.045% | 0.022% |
| **6** | *Lewinella* | 3.35% | 3.82% | 2.18% | 0.00% | 0.00% | 4.339% | 7.861% |
| **6** | *Saprospira* | 0.00% | 0.00% | 0.00% | 0.00% | 0.00% | 0.005% | 0.010% |
| **6** | unclassified | 51.39% | 40.90% | 17.08% | 1.22% | 0.42% | 64.699% | 19.536% |
| **5** | Chitinophagaceae | 0.18% | 0.51% | 0.45% | 0.00% | 0.00% | 0.023% | 0.034% |
| **6** | unclassified | 0.18% | 0.51% | 0.45% | 0.00% | 0.00% | 0.023% | 0.034% |
| **5** | Cytophagaceae | 0.02% | 0.00% | 0.00% | 0.00% | 0.00% | 0.042% | 0.080% |
| **6** | unclassified | 0.02% | 0.00% | 0.00% | 0.00% | 0.00% | 0.042% | 0.080% |
| **5** | unclassified | 7.47% | 14.78% | 6.66% | 0.07% | 0.04% | 4.411% | 0.532% |
| **6** | unclassified | 7.47% | 14.78% | 6.66% | 0.07% | 0.04% | 4.411% | 0.532% |
| **3** | Flavobacteria | 9.59% | 4.12% | 3.84% | 79.23% | 4.78% | 0.630% | 0.515% |
| **4** | Flavobacteriales | 9.59% | 4.12% | 3.84% | 79.23% | 4.78% | 0.630% | 0.515% |
| **5** | Cryomorphaceae | 0.00% | 0.00% | 0.00% | 0.00% | 0.00% | 0.005% | 0.012% |
| **6** | unclassified | 0.00% | 0.00% | 0.00% | 0.00% | 0.00% | 0.005% | 0.012% |
| **5** | Flavobacteriaceae | 9.59% | 4.11% | 3.83% | 79.23% | 4.78% | 0.625% | 0.519% |
| **6** | *Kordia* | 0.00% | 0.00% | 0.00% | 0.05% | 0.07% | 0.000% | 0.000% |
| **6** | *Krokinobacter* | 0.01% | 0.02% | 0.02% | 0.00% | 0.00% | 0.000% | 0.000% |
| **6** | *Maribacter* | 0.00% | 0.00% | 0.00% | 0.05% | 0.00% | 0.000% | 0.000% |
| **6** | *Tenacibaculum* | 0.02% | 0.04% | 0.04% | 0.00% | 0.00% | 0.009% | 0.013% |
| **6** | unclassified | 9.55% | 4.05% | 3.81% | 79.13% | 4.84% | 0.615% | 0.519% |
| **5** | unclassified | 0.00% | 0.01% | 0.02% | 0.00% | 0.00% | 0.000% | 0.000% |
| **6** | unclassified | 0.00% | 0.01% | 0.02% | 0.00% | 0.00% | 0.000% | 0.000% |
| **3** | unclassified | 4.90% | 6.17% | 3.78% | 0.32% | 0.06% | 4.830% | 1.490% |
| **4** | unclassified | 4.90% | 6.17% | 3.78% | 0.32% | 0.06% | 4.830% | 1.490% |
| **5** | unclassified | 4.90% | 6.17% | 3.78% | 0.32% | 0.06% | 4.830% | 1.490% |
| **6** | unclassified | 4.90% | 6.17% | 3.78% | 0.32% | 0.06% | 4.830% | 1.490% |
| **2** | Chloroflexi | 1.14% | 1.37% | 2.07% | 0.05% | 0.07% | 1.071% | 1.725% |
| **3** | unclassified | 1.14% | 1.37% | 2.07% | 0.05% | 0.07% | 1.071% | 1.725% |
| **4** | unclassified | 1.14% | 1.37% | 2.07% | 0.05% | 0.07% | 1.071% | 1.725% |
| **5** | unclassified | 1.14% | 1.37% | 2.07% | 0.05% | 0.07% | 1.071% | 1.725% |
| **6** | unclassified | 1.14% | 1.37% | 2.07% | 0.05% | 0.07% | 1.071% | 1.725% |
| **2** | Deinococcus-Thermus | 0.00% | 0.01% | 0.01% | 0.00% | 0.00% | 0.000% | 0.000% |
| **3** | Deinococci | 0.00% | 0.01% | 0.01% | 0.00% | 0.00% | 0.000% | 0.000% |
| **4** | Deinococcales | 0.00% | 0.01% | 0.01% | 0.00% | 0.00% | 0.000% | 0.000% |
| **5** | unclassified | 0.00% | 0.01% | 0.01% | 0.00% | 0.00% | 0.000% | 0.000% |
| **6** | unclassified | 0.00% | 0.01% | 0.01% | 0.00% | 0.00% | 0.000% | 0.000% |
| **2** | Planctomycetes | 0.78% | 1.11% | 1.39% | 4.06% | 1.22% | 0.043% | 0.059% |
| **3** | Planctomycetacia | 0.77% | 1.10% | 1.37% | 4.06% | 1.22% | 0.028% | 0.045% |
| **4** | Planctomycetales | 0.77% | 1.10% | 1.37% | 4.06% | 1.22% | 0.028% | 0.045% |
| **5** | Planctomycetaceae | 0.77% | 1.10% | 1.37% | 4.06% | 1.22% | 0.028% | 0.045% |
| **6** | *Rhodopirellula* | 0.34% | 1.03% | 1.29% | 0.02% | 0.03% | 0.024% | 0.036% |
| **6** | unclassified | 0.43% | 0.07% | 0.09% | 4.03% | 1.19% | 0.004% | 0.009% |
| **3** | Phycisphaerae | 0.01% | 0.00% | 0.00% | 0.00% | 0.00% | 0.015% | 0.033% |
| **4** | Phycisphaerales | 0.01% | 0.00% | 0.00% | 0.00% | 0.00% | 0.015% | 0.033% |
| **5** | Phycisphaeraceae | 0.01% | 0.00% | 0.00% | 0.00% | 0.00% | 0.015% | 0.033% |
| **6** | *Phycisphaera* | 0.01% | 0.00% | 0.00% | 0.00% | 0.00% | 0.015% | 0.033% |
| **3** | unclassified | 0.00% | 0.01% | 0.02% | 0.00% | 0.00% | 0.000% | 0.000% |
| **4** | unclassified | 0.00% | 0.01% | 0.02% | 0.00% | 0.00% | 0.000% | 0.000% |
| **5** | unclassified | 0.00% | 0.01% | 0.02% | 0.00% | 0.00% | 0.000% | 0.000% |
| **6** | unclassified | 0.00% | 0.01% | 0.02% | 0.00% | 0.00% | 0.000% | 0.000% |
| **2** | Proteobacteria | 11.16% | 9.02% | 4.55% | 14.17% | 3.44% | 13.093% | 15.783% |
| **3** | Alphaproteobacteria | 8.65% | 7.12% | 4.49% | 7.96% | 1.36% | 10.893% | 16.653% |
| **4** | Caulobacterales | 1.61% | 0.73% | 0.49% | 1.74% | 1.51% | 2.489% | 4.971% |
| **5** | Hyphomonadaceae | 1.51% | 0.57% | 0.39% | 1.74% | 1.51% | 2.418% | 4.861% |
| **6** | *Hellea* | 0.49% | 0.32% | 0.28% | 1.65% | 1.38% | 0.414% | 0.550% |
| **6** | *Robiginitomaculum* | 0.02% | 0.06% | 0.05% | 0.00% | 0.00% | 0.000% | 0.000% |
| **6** | unclassified | 1.00% | 0.20% | 0.18% | 0.09% | 0.13% | 2.004% | 4.321% |
| **5** | unclassified | 0.10% | 0.16% | 0.12% | 0.00% | 0.00% | 0.070% | 0.112% |
| **6** | unclassified | 0.10% | 0.16% | 0.12% | 0.00% | 0.00% | 0.070% | 0.112% |
| **4** | Rhizobiales | 0.01% | 0.02% | 0.03% | 0.00% | 0.00% | 0.000% | 0.000% |
| **5** | unclassified | 0.01% | 0.02% | 0.03% | 0.00% | 0.00% | 0.000% | 0.000% |
| **6** | unclassified | 0.01% | 0.02% | 0.03% | 0.00% | 0.00% | 0.000% | 0.000% |
| **4** | Rhodobacterales | 0.40% | 0.12% | 0.06% | 3.14% | 0.08% | 0.070% | 0.106% |
| **5** | Rhodobacteraceae | 0.40% | 0.12% | 0.06% | 3.14% | 0.08% | 0.070% | 0.106% |
| **6** | *Loktanella* | 0.00% | 0.01% | 0.02% | 0.00% | 0.00% | 0.000% | 0.000% |
| **6** | *Roseovarius* | 0.01% | 0.01% | 0.02% | 0.00% | 0.00% | 0.007% | 0.017% |
| **6** | *Sulfitobacter* | 0.00% | 0.00% | 0.00% | 0.00% | 0.00% | 0.009% | 0.021% |
| **6** | unclassified | 0.38% | 0.10% | 0.04% | 3.14% | 0.08% | 0.053% | 0.087% |
| **4** | Sphingomonadales | 0.31% | 0.01% | 0.02% | 2.85% | 0.19% | 0.040% | 0.039% |
| **5** | Erythrobacteraceae | 0.00% | 0.01% | 0.01% | 0.00% | 0.00% | 0.000% | 0.000% |
| **6** | *Altererythrobacter* | 0.00% | 0.01% | 0.01% | 0.00% | 0.00% | 0.000% | 0.000% |
| **5** | Sphingomonadaceae | 0.30% | 0.00% | 0.00% | 2.80% | 0.19% | 0.031% | 0.043% |
| **6** | *Sphingopyxis* | 0.29% | 0.00% | 0.00% | 2.70% | 0.11% | 0.031% | 0.043% |
| **6** | unclassified | 0.01% | 0.00% | 0.00% | 0.10% | 0.08% | 0.000% | 0.000% |
| **5** | unclassified | 0.01% | 0.01% | 0.02% | 0.05% | 0.00% | 0.009% | 0.021% |
| **6** | unclassified | 0.01% | 0.01% | 0.02% | 0.05% | 0.00% | 0.009% | 0.021% |
| **4** | unclassified | 6.33% | 6.23% | 4.00% | 0.24% | 0.12% | 8.294% | 11.845% |
| **5** | unclassified | 6.33% | 6.23% | 4.00% | 0.24% | 0.12% | 8.294% | 11.845% |
| **6** | unclassified | 6.33% | 6.23% | 4.00% | 0.24% | 0.12% | 8.294% | 11.845% |
| **3** | Betaproteobacteria | 0.00% | 0.00% | 0.00% | 0.00% | 0.00% | 0.005% | 0.010% |
| **4** | Burkholderiales | 0.00% | 0.00% | 0.00% | 0.00% | 0.00% | 0.005% | 0.010% |
| **5** | Oxalobacteraceae | 0.00% | 0.00% | 0.00% | 0.00% | 0.00% | 0.005% | 0.010% |
| **6** | unclassified | 0.00% | 0.00% | 0.00% | 0.00% | 0.00% | 0.005% | 0.010% |
| **3** | Deltaproteobacteria | 0.01% | 0.04% | 0.05% | 0.00% | 0.00% | 0.000% | 0.000% |
| **4** | Bdellovibrionales | 0.01% | 0.03% | 0.03% | 0.00% | 0.00% | 0.000% | 0.000% |
| **5** | Bacteriovoracaceae | 0.01% | 0.03% | 0.03% | 0.00% | 0.00% | 0.000% | 0.000% |
| **6** | *Bacteriovorax* | 0.01% | 0.03% | 0.03% | 0.00% | 0.00% | 0.000% | 0.000% |
| **4** | unclassified | 0.00% | 0.02% | 0.02% | 0.00% | 0.00% | 0.000% | 0.000% |
| **5** | unclassified | 0.00% | 0.02% | 0.02% | 0.00% | 0.00% | 0.000% | 0.000% |
| **6** | unclassified | 0.00% | 0.02% | 0.02% | 0.00% | 0.00% | 0.000% | 0.000% |
| **3** | Gammaproteobacteria | 2.24% | 1.52% | 0.38% | 6.14% | 1.99% | 1.940% | 2.047% |
| **4** | Alteromonadales | 0.63% | 0.11% | 0.15% | 5.66% | 1.89% | 0.038% | 0.039% |
| **5** | Alteromonadaceae | 0.57% | 0.00% | 0.00% | 5.66% | 1.89% | 0.008% | 0.018% |
| **6** | *Aestuariibacter* | 0.09% | 0.00% | 0.00% | 0.89% | 0.35% | 0.004% | 0.009% |
| **6** | unclassified | 0.48% | 0.00% | 0.00% | 4.77% | 2.23% | 0.004% | 0.009% |
| **5** | Colwelliaceae | 0.02% | 0.06% | 0.10% | 0.00% | 0.00% | 0.000% | 0.000% |
| **6** | unclassified | 0.02% | 0.06% | 0.10% | 0.00% | 0.00% | 0.000% | 0.000% |
| **5** | Moritellaceae | 0.00% | 0.00% | 0.00% | 0.00% | 0.00% | 0.005% | 0.012% |
| **6** | *Moritella* | 0.00% | 0.00% | 0.00% | 0.00% | 0.00% | 0.005% | 0.012% |
| **5** | Pseudoalteromonadaceae | 0.01% | 0.03% | 0.06% | 0.00% | 0.00% | 0.005% | 0.012% |
| **6** | *Pseudoalteromonas* | 0.01% | 0.03% | 0.06% | 0.00% | 0.00% | 0.005% | 0.012% |
| **5** | Psychromonadaceae | 0.01% | 0.01% | 0.02% | 0.00% | 0.00% | 0.019% | 0.042% |
| **6** | *Psychromonas* | 0.01% | 0.01% | 0.02% | 0.00% | 0.00% | 0.019% | 0.042% |
| **5** | unclassified | 0.00% | 0.01% | 0.02% | 0.00% | 0.00% | 0.000% | 0.000% |
| **6** | unclassified | 0.00% | 0.01% | 0.02% | 0.00% | 0.00% | 0.000% | 0.000% |
| **4** | Chromatiales | 1.12% | 0.50% | 0.24% | 0.05% | 0.07% | 1.654% | 1.926% |
| **5** | Granulosicoccaceae | 1.08% | 0.46% | 0.23% | 0.05% | 0.07% | 1.620% | 1.895% |
| **6** | *Granulosicoccus* | 1.08% | 0.46% | 0.23% | 0.05% | 0.07% | 1.620% | 1.895% |
| **5** | unclassified | 0.03% | 0.04% | 0.05% | 0.00% | 0.00% | 0.034% | 0.032% |
| **6** | unclassified | 0.03% | 0.04% | 0.05% | 0.00% | 0.00% | 0.034% | 0.032% |
| **4** | Gammaproteobacteria_incertae_sedis | 0.00% | 0.01% | 0.02% | 0.00% | 0.00% | 0.005% | 0.010% |
| **5** | *Arenicella* | 0.00% | 0.00% | 0.00% | 0.00% | 0.00% | 0.005% | 0.010% |
| **6** | unclassified | 0.00% | 0.00% | 0.00% | 0.00% | 0.00% | 0.005% | 0.010% |
| **5** | *Cocleimonas* | 0.00% | 0.01% | 0.02% | 0.00% | 0.00% | 0.000% | 0.000% |
| **6** | unclassified | 0.00% | 0.01% | 0.02% | 0.00% | 0.00% | 0.000% | 0.000% |
| **4** | Oceanospirillales | 0.00% | 0.01% | 0.01% | 0.00% | 0.00% | 0.000% | 0.000% |
| **5** | Oceanospirillaceae | 0.00% | 0.01% | 0.01% | 0.00% | 0.00% | 0.000% | 0.000% |
| **6** | *Neptunomonas* | 0.00% | 0.01% | 0.01% | 0.00% | 0.00% | 0.000% | 0.000% |
| **4** | Pseudomonadales | 0.03% | 0.00% | 0.00% | 0.24% | 0.05% | 0.005% | 0.012% |
| **5** | Moraxellaceae | 0.00% | 0.00% | 0.00% | 0.00% | 0.00% | 0.005% | 0.012% |
| **6** | *Acinetobacter* | 0.00% | 0.00% | 0.00% | 0.00% | 0.00% | 0.005% | 0.012% |
| **5** | Pseudomonadaceae | 0.02% | 0.00% | 0.00% | 0.24% | 0.05% | 0.000% | 0.000% |
| **6** | unclassified | 0.02% | 0.00% | 0.00% | 0.24% | 0.05% | 0.000% | 0.000% |
| **4** | unclassified | 0.46% | 0.90% | 0.15% | 0.19% | 0.02% | 0.239% | 0.169% |
| **5** | unclassified | 0.46% | 0.90% | 0.15% | 0.19% | 0.02% | 0.239% | 0.169% |
| **6** | unclassified | 0.46% | 0.90% | 0.15% | 0.19% | 0.02% | 0.239% | 0.169% |
| **3** | unclassified | 0.26% | 0.34% | 0.05% | 0.07% | 0.10% | 0.255% | 0.137% |
| **4** | unclassified | 0.26% | 0.34% | 0.05% | 0.07% | 0.10% | 0.255% | 0.137% |
| **5** | unclassified | 0.26% | 0.34% | 0.05% | 0.07% | 0.10% | 0.255% | 0.137% |
| **6** | unclassified | 0.26% | 0.34% | 0.05% | 0.07% | 0.10% | 0.255% | 0.137% |
| **2** | Firmicutes | 0.00% | 0.00% | 0.00% | 0.00% | 0.00% | 0.004% | 0.009% |
| **3** | Bacilli | 0.00% | 0.00% | 0.00% | 0.00% | 0.00% | 0.004% | 0.009% |
| **4** | Bacillales | 0.00% | 0.00% | 0.00% | 0.00% | 0.00% | 0.004% | 0.009% |
| **5** | unclassified | 0.00% | 0.00% | 0.00% | 0.00% | 0.00% | 0.004% | 0.009% |
| **6** | unclassified | 0.00% | 0.00% | 0.00% | 0.00% | 0.00% | 0.004% | 0.009% |
| **2** | unclassified | 9.26% | 16.30% | 6.86% | 0.89% | 0.28% | 6.559% | 2.059% |
| **3** | unclassified | 9.26% | 16.30% | 6.86% | 0.89% | 0.28% | 6.559% | 2.059% |
| **4** | unclassified | 9.26% | 16.30% | 6.86% | 0.89% | 0.28% | 6.559% | 2.059% |
| **5** | unclassified | 9.26% | 16.30% | 6.86% | 0.89% | 0.28% | 6.559% | 2.059% |
| **6** | unclassified | 9.26% | 16.30% | 6.86% | 0.89% | 0.28% | 6.559% | 2.059% |

Table S3. Distribution of OTUs at 0.03 distance formed from V8 data (41,514 sequences). A total of 2526 OTUs were formed across all samples.

| **Tax level** | **Taxon** | **Total** | **F1** | **F2** | **F3** | **F4** | **F5** | **L1** | **L2** | **W1** | **W2** | **W3** | **W4** | **W5** |
| --- | --- | --- | --- | --- | --- | --- | --- | --- | --- | --- | --- | --- | --- | --- |
| 0 | Root | 2526 | 448 | 381 | 438 | 336 | 462 | 79 | 74 | 336 | 315 | 182 | 269 | 420 |
| 1 | Bacteria | 2526 | 448 | 381 | 438 | 336 | 462 | 79 | 74 | 336 | 315 | 182 | 269 | 420 |
| 2 | Actinobacteria | 22 | 10 | 0 | 5 | 1 | 4 | 0 | 0 | 2 | 0 | 0 | 0 | 8 |
| 3 | Actinobacteria | 22 | 10 | 0 | 5 | 1 | 4 | 0 | 0 | 2 | 0 | 0 | 0 | 8 |
| 4 | Acidimicrobiales | 7 | 5 | 0 | 2 | 1 | 2 | 0 | 0 | 1 | 0 | 0 | 0 | 3 |
| 5 | Acidimicrobiaceae | 2 | 2 | 0 | 0 | 0 | 1 | 0 | 0 | 1 | 0 | 0 | 0 | 1 |
| 6 | *Ilumatobacter* | 1 | 1 | 0 | 0 | 0 | 1 | 0 | 0 | 1 | 0 | 0 | 0 | 1 |
| 6 | unclassified | 1 | 1 | 0 | 0 | 0 | 0 | 0 | 0 | 0 | 0 | 0 | 0 | 0 |
| 5 | Iamiaceae | 1 | 0 | 0 | 1 | 0 | 0 | 0 | 0 | 0 | 0 | 0 | 0 | 0 |
| 6 | *Iamia* | 1 | 0 | 0 | 1 | 0 | 0 | 0 | 0 | 0 | 0 | 0 | 0 | 0 |
| 5 | unclassified | 4 | 3 | 0 | 1 | 1 | 1 | 0 | 0 | 0 | 0 | 0 | 0 | 2 |
| 6 | unclassified | 4 | 3 | 0 | 1 | 1 | 1 | 0 | 0 | 0 | 0 | 0 | 0 | 2 |
| 4 | unclassified | 15 | 5 | 0 | 3 | 0 | 2 | 0 | 0 | 1 | 0 | 0 | 0 | 5 |
| 5 | unclassified | 15 | 5 | 0 | 3 | 0 | 2 | 0 | 0 | 1 | 0 | 0 | 0 | 5 |
| 6 | unclassified | 15 | 5 | 0 | 3 | 0 | 2 | 0 | 0 | 1 | 0 | 0 | 0 | 5 |
| 2 | Bacteroidetes | 866 | 173 | 135 | 176 | 157 | 226 | 32 | 34 | 120 | 73 | 52 | 100 | 162 |
| 3 | Sphingobacteria | 548 | 135 | 106 | 128 | 117 | 161 | 2 | 3 | 80 | 47 | 40 | 58 | 96 |
| 4 | Sphingobacteriales | 548 | 135 | 106 | 128 | 117 | 161 | 2 | 3 | 80 | 47 | 40 | 58 | 96 |
| 5 | Saprospiraceae | 252 | 70 | 53 | 67 | 61 | 80 | 2 | 3 | 35 | 27 | 13 | 22 | 44 |
| 6 | *Aureispira* | 3 | 2 | 1 | 2 | 1 | 1 | 0 | 0 | 1 | 0 | 0 | 1 | 2 |
| 6 | *Haliscomenobacter* | 10 | 2 | 3 | 3 | 0 | 7 | 0 | 0 | 1 | 1 | 1 | 1 | 1 |
| 6 | *Lewinella* | 66 | 18 | 13 | 13 | 9 | 23 | 0 | 0 | 15 | 16 | 2 | 3 | 6 |
| 6 | *Saprospira* | 1 | 0 | 0 | 0 | 0 | 0 | 0 | 0 | 1 | 0 | 0 | 0 | 0 |
| 6 | unclassified | 172 | 48 | 36 | 49 | 50 | 49 | 2 | 3 | 17 | 10 | 10 | 17 | 35 |
| 5 | Chitinophagaceae | 4 | 2 | 0 | 2 | 0 | 0 | 0 | 0 | 0 | 0 | 0 | 0 | 0 |
| 6 | unclassified | 4 | 2 | 0 | 2 | 0 | 0 | 0 | 0 | 0 | 0 | 0 | 0 | 0 |
| 5 | Cytophagaceae | 1 | 0 | 0 | 0 | 0 | 0 | 0 | 0 | 1 | 1 | 0 | 1 | 0 |
| 6 | unclassified | 1 | 0 | 0 | 0 | 0 | 0 | 0 | 0 | 1 | 1 | 0 | 1 | 0 |
| 5 | unclassified | 291 | 63 | 53 | 59 | 56 | 81 | 0 | 0 | 44 | 19 | 27 | 35 | 52 |
| 6 | unclassified | 291 | 63 | 53 | 59 | 56 | 81 | 0 | 0 | 44 | 19 | 27 | 35 | 52 |
| 3 | Flavobacteria | 109 | 15 | 4 | 7 | 8 | 20 | 27 | 29 | 9 | 7 | 1 | 7 | 18 |
| 4 | Flavobacteriales | 109 | 15 | 4 | 7 | 8 | 20 | 27 | 29 | 9 | 7 | 1 | 7 | 18 |
| 5 | Cryomorphaceae | 1 | 0 | 0 | 0 | 0 | 0 | 0 | 0 | 0 | 1 | 0 | 0 | 0 |
| 6 | unclassified | 1 | 0 | 0 | 0 | 0 | 0 | 0 | 0 | 0 | 1 | 0 | 0 | 0 |
| 5 | Flavobacteriaceae | 107 | 15 | 4 | 7 | 8 | 19 | 27 | 29 | 9 | 6 | 1 | 7 | 18 |
| 6 | *Krokinobacter* | 1 | 1 | 1 | 0 | 0 | 1 | 0 | 0 | 0 | 0 | 0 | 0 | 0 |
| 6 | *Tenacibaculum* | 1 | 0 | 0 | 1 | 1 | 1 | 0 | 0 | 0 | 1 | 0 | 1 | 0 |
| 6 | unclassified | 105 | 14 | 3 | 6 | 7 | 17 | 27 | 29 | 9 | 5 | 1 | 6 | 18 |
| 5 | unclassified | 1 | 0 | 0 | 0 | 0 | 1 | 0 | 0 | 0 | 0 | 0 | 0 | 0 |
| 6 | unclassified | 1 | 0 | 0 | 0 | 0 | 1 | 0 | 0 | 0 | 0 | 0 | 0 | 0 |
| 3 | unclassified | 209 | 23 | 25 | 41 | 32 | 45 | 3 | 2 | 31 | 19 | 11 | 35 | 48 |
| 4 | unclassified | 209 | 23 | 25 | 41 | 32 | 45 | 3 | 2 | 31 | 19 | 11 | 35 | 48 |
| 5 | unclassified | 209 | 23 | 25 | 41 | 32 | 45 | 3 | 2 | 31 | 19 | 11 | 35 | 48 |
| 6 | unclassified | 209 | 23 | 25 | 41 | 32 | 45 | 3 | 2 | 31 | 19 | 11 | 35 | 48 |
| 2 | Chloroflexi | 16 | 7 | 3 | 2 | 2 | 5 | 1 | 0 | 4 | 0 | 2 | 3 | 8 |
| 3 | unclassified | 16 | 7 | 3 | 2 | 2 | 5 | 1 | 0 | 4 | 0 | 2 | 3 | 8 |
| 4 | unclassified | 16 | 7 | 3 | 2 | 2 | 5 | 1 | 0 | 4 | 0 | 2 | 3 | 8 |
| 5 | unclassified | 16 | 7 | 3 | 2 | 2 | 5 | 1 | 0 | 4 | 0 | 2 | 3 | 8 |
| 6 | unclassified | 16 | 7 | 3 | 2 | 2 | 5 | 1 | 0 | 4 | 0 | 2 | 3 | 8 |
| 2 | Planctomycetes | 24 | 9 | 2 | 7 | 0 | 2 | 4 | 3 | 0 | 0 | 0 | 3 | 6 |
| 3 | Planctomycetacia | 19 | 8 | 2 | 7 | 0 | 2 | 4 | 3 | 0 | 0 | 0 | 3 | 2 |
| 4 | Planctomycetales | 19 | 8 | 2 | 7 | 0 | 2 | 4 | 3 | 0 | 0 | 0 | 3 | 2 |
| 5 | Planctomycetaceae | 19 | 8 | 2 | 7 | 0 | 2 | 4 | 3 | 0 | 0 | 0 | 3 | 2 |
| 6 | *Rhodopirellula* | 12 | 7 | 2 | 7 | 0 | 2 | 1 | 0 | 0 | 0 | 0 | 2 | 2 |
| 6 | unclassified | 7 | 1 | 0 | 0 | 0 | 0 | 3 | 3 | 0 | 0 | 0 | 1 | 0 |
| 3 | Phycisphaerae | 4 | 0 | 0 | 0 | 0 | 0 | 0 | 0 | 0 | 0 | 0 | 0 | 4 |
| 4 | Phycisphaerales | 4 | 0 | 0 | 0 | 0 | 0 | 0 | 0 | 0 | 0 | 0 | 0 | 4 |
| 5 | Phycisphaeraceae | 4 | 0 | 0 | 0 | 0 | 0 | 0 | 0 | 0 | 0 | 0 | 0 | 4 |
| 6 | *Phycisphaera* | 4 | 0 | 0 | 0 | 0 | 0 | 0 | 0 | 0 | 0 | 0 | 0 | 4 |
| 3 | unclassified | 1 | 1 | 0 | 0 | 0 | 0 | 0 | 0 | 0 | 0 | 0 | 0 | 0 |
| 4 | unclassified | 1 | 1 | 0 | 0 | 0 | 0 | 0 | 0 | 0 | 0 | 0 | 0 | 0 |
| 5 | unclassified | 1 | 1 | 0 | 0 | 0 | 0 | 0 | 0 | 0 | 0 | 0 | 0 | 0 |
| 6 | unclassified | 1 | 1 | 0 | 0 | 0 | 0 | 0 | 0 | 0 | 0 | 0 | 0 | 0 |
| 2 | Proteobacteria | 510 | 92 | 118 | 69 | 59 | 42 | 31 | 20 | 91 | 140 | 35 | 43 | 53 |
| 3 | Alphaproteobacteria | 337 | 75 | 90 | 51 | 40 | 23 | 22 | 12 | 74 | 111 | 15 | 7 | 40 |
| 4 | Caulobacterales | 47 | 11 | 7 | 5 | 5 | 3 | 9 | 1 | 5 | 20 | 2 | 3 | 4 |
| 5 | Hyphomonadaceae | 43 | 9 | 5 | 3 | 4 | 3 | 9 | 1 | 4 | 19 | 2 | 3 | 3 |
| 6 | *Hellea* | 16 | 5 | 0 | 0 | 1 | 0 | 5 | 0 | 0 | 5 | 0 | 2 | 0 |
| 6 | *Robiginitomaculum* | 2 | 0 | 0 | 0 | 0 | 2 | 0 | 0 | 0 | 0 | 0 | 0 | 0 |
| 6 | unclassified | 25 | 4 | 5 | 3 | 3 | 1 | 4 | 1 | 4 | 14 | 2 | 1 | 3 |
| 5 | unclassified | 4 | 2 | 2 | 2 | 1 | 0 | 0 | 0 | 1 | 1 | 0 | 0 | 1 |
| 6 | unclassified | 4 | 2 | 2 | 2 | 1 | 0 | 0 | 0 | 1 | 1 | 0 | 0 | 1 |
| 4 | Rhodobacterales | 21 | 1 | 7 | 2 | 2 | 2 | 5 | 5 | 6 | 0 | 1 | 0 | 2 |
| 5 | Rhodobacteraceae | 21 | 1 | 7 | 2 | 2 | 2 | 5 | 5 | 6 | 0 | 1 | 0 | 2 |
| 6 | *Loktanella* | 1 | 0 | 1 | 0 | 0 | 0 | 0 | 0 | 0 | 0 | 0 | 0 | 0 |
| 6 | unclassified | 20 | 1 | 6 | 2 | 2 | 2 | 5 | 5 | 6 | 0 | 1 | 0 | 2 |
| 4 | Sphingomonadales | 8 | 0 | 1 | 1 | 0 | 0 | 4 | 3 | 1 | 0 | 0 | 1 | 1 |
| 5 | Erythrobacteraceae | 1 | 0 | 1 | 0 | 0 | 0 | 0 | 0 | 0 | 0 | 0 | 0 | 0 |
| 6 | Altererythrobacter | 1 | 0 | 1 | 0 | 0 | 0 | 0 | 0 | 0 | 0 | 0 | 0 | 0 |
| 5 | Sphingomonadaceae | 4 | 0 | 0 | 0 | 0 | 0 | 3 | 2 | 0 | 0 | 0 | 1 | 1 |
| 6 | *Sphingopyxis* | 3 | 0 | 0 | 0 | 0 | 0 | 3 | 1 | 0 | 0 | 0 | 1 | 1 |
| 6 | unclassified | 1 | 0 | 0 | 0 | 0 | 0 | 0 | 1 | 0 | 0 | 0 | 0 | 0 |
| 5 | unclassified | 3 | 0 | 0 | 1 | 0 | 0 | 1 | 1 | 1 | 0 | 0 | 0 | 0 |
| 6 | unclassified | 3 | 0 | 0 | 1 | 0 | 0 | 1 | 1 | 1 | 0 | 0 | 0 | 0 |
| 4 | unclassified | 261 | 63 | 75 | 43 | 33 | 18 | 4 | 3 | 62 | 91 | 12 | 3 | 33 |
| 5 | unclassified | 261 | 63 | 75 | 43 | 33 | 18 | 4 | 3 | 62 | 91 | 12 | 3 | 33 |
| 6 | unclassified | 261 | 63 | 75 | 43 | 33 | 18 | 4 | 3 | 62 | 91 | 12 | 3 | 33 |
| 3 | Betaproteobacteria | 1 | 0 | 0 | 0 | 0 | 0 | 0 | 0 | 0 | 0 | 1 | 0 | 0 |
| 4 | Burkholderiales | 1 | 0 | 0 | 0 | 0 | 0 | 0 | 0 | 0 | 0 | 1 | 0 | 0 |
| 5 | Oxalobacteraceae | 1 | 0 | 0 | 0 | 0 | 0 | 0 | 0 | 0 | 0 | 1 | 0 | 0 |
| 6 | unclassified | 1 | 0 | 0 | 0 | 0 | 0 | 0 | 0 | 0 | 0 | 1 | 0 | 0 |
| 3 | Deltaproteobacteria | 4 | 0 | 0 | 1 | 1 | 3 | 0 | 0 | 0 | 0 | 0 | 0 | 0 |
| 4 | Bdellovibrionales | 2 | 0 | 0 | 0 | 1 | 2 | 0 | 0 | 0 | 0 | 0 | 0 | 0 |
| 5 | Bacteriovoracaceae | 2 | 0 | 0 | 0 | 1 | 2 | 0 | 0 | 0 | 0 | 0 | 0 | 0 |
| 6 | Bacteriovorax | 2 | 0 | 0 | 0 | 1 | 2 | 0 | 0 | 0 | 0 | 0 | 0 | 0 |
| 4 | unclassified | 2 | 0 | 0 | 1 | 0 | 1 | 0 | 0 | 0 | 0 | 0 | 0 | 0 |
| 5 | unclassified | 2 | 0 | 0 | 1 | 0 | 1 | 0 | 0 | 0 | 0 | 0 | 0 | 0 |
| 6 | unclassified | 2 | 0 | 0 | 1 | 0 | 1 | 0 | 0 | 0 | 0 | 0 | 0 | 0 |
| 3 | Gammaproteobacteria | 96 | 9 | 17 | 7 | 13 | 9 | 7 | 8 | 6 | 15 | 16 | 28 | 8 |
| 4 | Alteromonadales | 11 | 1 | 2 | 0 | 2 | 1 | 2 | 5 | 0 | 2 | 1 | 1 | 1 |
| 5 | Alteromonadaceae | 6 | 0 | 0 | 0 | 0 | 0 | 2 | 5 | 0 | 0 | 0 | 1 | 1 |
| 6 | *Aestuariibacter* | 2 | 0 | 0 | 0 | 0 | 0 | 0 | 2 | 0 | 0 | 0 | 0 | 0 |
| 6 | unclassified | 4 | 0 | 0 | 0 | 0 | 0 | 2 | 3 | 0 | 0 | 0 | 1 | 1 |
| 5 | Colwelliaceae | 1 | 1 | 1 | 0 | 1 | 1 | 0 | 0 | 0 | 0 | 0 | 0 | 0 |
| 6 | unclassified | 1 | 1 | 1 | 0 | 1 | 1 | 0 | 0 | 0 | 0 | 0 | 0 | 0 |
| 5 | Moritellaceae | 1 | 0 | 0 | 0 | 0 | 0 | 0 | 0 | 0 | 1 | 0 | 0 | 0 |
| 6 | *Moritella* | 1 | 0 | 0 | 0 | 0 | 0 | 0 | 0 | 0 | 1 | 0 | 0 | 0 |
| 5 | Pseudoalteromonadaceae | 1 | 0 | 0 | 0 | 1 | 0 | 0 | 0 | 0 | 1 | 0 | 0 | 0 |
| 6 | Pseudoalteromonas | 1 | 0 | 0 | 0 | 1 | 0 | 0 | 0 | 0 | 1 | 0 | 0 | 0 |
| 5 | Psychromonadaceae | 2 | 0 | 1 | 0 | 0 | 0 | 0 | 0 | 0 | 0 | 1 | 0 | 0 |
| 6 | Psychromonas | 2 | 0 | 1 | 0 | 0 | 0 | 0 | 0 | 0 | 0 | 1 | 0 | 0 |
| 4 | Chromatiales | 24 | 2 | 2 | 1 | 5 | 2 | 2 | 0 | 4 | 2 | 6 | 12 | 3 |
| 5 | Granulosicoccaceae | 20 | 2 | 2 | 1 | 3 | 2 | 2 | 0 | 4 | 2 | 5 | 10 | 3 |
| 6 | *Granulosicoccus* | 20 | 2 | 2 | 1 | 3 | 2 | 2 | 0 | 4 | 2 | 5 | 10 | 3 |
| 5 | unclassified | 4 | 0 | 0 | 0 | 2 | 0 | 0 | 0 | 0 | 0 | 1 | 2 | 0 |
| 6 | unclassified | 4 | 0 | 0 | 0 | 2 | 0 | 0 | 0 | 0 | 0 | 1 | 2 | 0 |
| 4 | Gammaproteobacteria_incertae_sedis | 2 | 1 | 0 | 0 | 0 | 0 | 0 | 0 | 0 | 0 | 1 | 0 | 0 |
| 5 | *Arenicella* | 1 | 0 | 0 | 0 | 0 | 0 | 0 | 0 | 0 | 0 | 1 | 0 | 0 |
| 6 | unclassified | 1 | 0 | 0 | 0 | 0 | 0 | 0 | 0 | 0 | 0 | 1 | 0 | 0 |
| 5 | Cocleimonas | 1 | 1 | 0 | 0 | 0 | 0 | 0 | 0 | 0 | 0 | 0 | 0 | 0 |
| 6 | unclassified | 1 | 1 | 0 | 0 | 0 | 0 | 0 | 0 | 0 | 0 | 0 | 0 | 0 |
| 4 | Oceanospirillales | 1 | 0 | 1 | 0 | 0 | 0 | 0 | 0 | 0 | 0 | 0 | 0 | 0 |
| 5 | Oceanospirillaceae | 1 | 0 | 1 | 0 | 0 | 0 | 0 | 0 | 0 | 0 | 0 | 0 | 0 |
| 6 | *Neptunomonas* | 1 | 0 | 1 | 0 | 0 | 0 | 0 | 0 | 0 | 0 | 0 | 0 | 0 |
| 4 | Pseudomonadales | 2 | 0 | 0 | 0 | 0 | 0 | 1 | 1 | 0 | 1 | 0 | 0 | 0 |
| 5 | Moraxellaceae | 1 | 0 | 0 | 0 | 0 | 0 | 0 | 0 | 0 | 1 | 0 | 0 | 0 |
| 6 | *Acinetobacter* | 1 | 0 | 0 | 0 | 0 | 0 | 0 | 0 | 0 | 1 | 0 | 0 | 0 |
| 5 | Pseudomonadaceae | 1 | 0 | 0 | 0 | 0 | 0 | 1 | 1 | 0 | 0 | 0 | 0 | 0 |
| 6 | unclassified | 1 | 0 | 0 | 0 | 0 | 0 | 1 | 1 | 0 | 0 | 0 | 0 | 0 |
| 4 | unclassified | 56 | 5 | 12 | 6 | 6 | 6 | 2 | 2 | 2 | 10 | 8 | 15 | 4 |
| 5 | unclassified | 56 | 5 | 12 | 6 | 6 | 6 | 2 | 2 | 2 | 10 | 8 | 15 | 4 |
| 6 | unclassified | 56 | 5 | 12 | 6 | 6 | 6 | 2 | 2 | 2 | 10 | 8 | 15 | 4 |
| 3 | unclassified | 72 | 8 | 11 | 10 | 5 | 7 | 2 | 0 | 11 | 14 | 3 | 8 | 5 |
| 4 | unclassified | 72 | 8 | 11 | 10 | 5 | 7 | 2 | 0 | 11 | 14 | 3 | 8 | 5 |
| 5 | unclassified | 72 | 8 | 11 | 10 | 5 | 7 | 2 | 0 | 11 | 14 | 3 | 8 | 5 |
| 6 | unclassified | 72 | 8 | 11 | 10 | 5 | 7 | 2 | 0 | 11 | 14 | 3 | 8 | 5 |
| 2 | Firmicutes | 1 | 0 | 0 | 0 | 0 | 0 | 0 | 0 | 0 | 0 | 0 | 1 | 0 |
| 3 | Bacilli | 1 | 0 | 0 | 0 | 0 | 0 | 0 | 0 | 0 | 0 | 0 | 1 | 0 |
| 4 | Bacillales | 1 | 0 | 0 | 0 | 0 | 0 | 0 | 0 | 0 | 0 | 0 | 1 | 0 |
| 5 | unclassified | 1 | 0 | 0 | 0 | 0 | 0 | 0 | 0 | 0 | 0 | 0 | 1 | 0 |
| 6 | unclassified | 1 | 0 | 0 | 0 | 0 | 0 | 0 | 0 | 0 | 0 | 0 | 1 | 0 |
| 2 | unclassified | 1087 | 157 | 123 | 179 | 117 | 183 | 11 | 17 | 119 | 102 | 93 | 119 | 183 |
| 3 | unclassified | 1087 | 157 | 123 | 179 | 117 | 183 | 11 | 17 | 119 | 102 | 93 | 119 | 183 |
| 4 | unclassified | 1087 | 157 | 123 | 179 | 117 | 183 | 11 | 17 | 119 | 102 | 93 | 119 | 183 |
| 5 | unclassified | 1087 | 157 | 123 | 179 | 117 | 183 | 11 | 17 | 119 | 102 | 93 | 119 | 183 |
| 6 | unclassified | 1087 | 157 | 123 | 179 | 117 | 183 | 11 | 17 | 119 | 102 | 93 | 119 | 183 |

Table S4. Species richness and diversity of the bacterial communities associated with *P. umbilicalis* based upon analysis of the V8 variable region. Mean values +/- 95% confidence interval are provided for Chao1 and the Inverse Simpson index.

| **I. OTUs formed at 0.03 distance^a^** |  |  |  |  |  |  |
| --- | --- | --- | --- | --- | --- | --- |
| **Blade ID** | **Observed**  **OTUs** | **Observed OTUs**  **(Normalized Subsample)** | **Chao1** | **Chao1(Normalized Subsample)** | **Inverse Simpson**  **Index** | **Inverse Simpson Index**  **Normalized Subsample** |
| Fall 1 | 448 | 367 | 1718 (1328,2281) | 1604 (1177, 2257) | 27.23 (25.25, 29.60) | 27.93 (25.55, 30.82) |
| Fall 2 | 381 | 219 | 1452 (1102,1971) | 618 (464, 869) | 2.44 (2.33, 2.56) | 2.46 (2.31, 2.63) |
| Fall 3 | 438 | 369 | 1714 (1317,2292) | 1341 (1018, 1823) | 9.25 (8.41, 10.28) | 9.35 (8.41, 10.53) |
| Fall 4 | 336 | 253 | 1006 (784, 1338) | 895 (646, 1300) | 5.79 (5.40 6.23) | 5.64 (5.19, 6.18) |
| Fall 5 | 462 | 344 | 1868 (1433,2497) | 1183 (906, 1596) | 20.63 (19.20, 22.29) | 20.0 (18.3, 22.0) |
| Winter 1 | 336 | 185 | 860 (695, 1101) | 590 (425, 868) | 2.40 (2.30, 2.50) | 1.99 (1.88, 2.10) |
| Winter 2 | 315 | 184 | 1155 (871, 1585) | 828 (548, 1321) | 5.70 (5.45, 5.98) | 5.81 (5.46, 6.21) |
| Winter 3 | 183 | 108 | 588 (420, 878) | 440 (270, 787) | 1.63 (1.58, 1.68) | 1.57 (1.51, 1.64) |
| Winter 4 | 270 | 133 | 929 (688, 1308) | 505 (324, 856) | 1.53 (1.49, 1.57) | 1.48 (1.42, 1.54) |
| Winter 5 | 420 | 185 | 1672 (1278,2247) | 834 (547, 1347) | 2.09 (2.02, 2.16) | 2.09 (1.97, 2.21) |
| Lab 1 | 79 | 74 | 222 (144, 392) | 196 (129, 346) | 2.38 (2.25, 2.52) | 2.35 (2.22, 2.49) |
| Lab 2 | 74 | 74 | 196 (129, 346) | 196 (129, 346) | 2.51 (2.38, 2.65) | 2.51 (2.39, 2.65) |
| II. **OTUs formed at 0.05 distance^b^** |  |  |  |  |  |  |
| **Blade**  **ID** | **Observed**  **OTUs** | **Observed OTUs**  **(Normalized**  **Subsample)** | **Chao1** | **Chao1**  **(Normalized Subsample)** | **Inverse Simpson**  **Index** | **Inverse Simpson Index**  **Normalized Subsample** |
| Fall 1 | 252 | 204 | 818 (602, 1167) | 670 (475, 1004) | 18.89 (17.82, 20.1) | 18.59 (17.34, 20.03) |
| Fall 2 | 221 | 140 | 503 (396, 676) | 431 (295, 687) | 2.36 (2.26, 2.5) | 2.4 (2.25, 2.56) |
| Fall 3 | 438 | 229 | 1714 (1317, 2292) | 875 (604, 1343) | 9.25 (8.41, 10.28) | 8.23 (7.49, 9.15) |
| Fall 4 | 183 | 144 | 398 (305, 562) | 307 (230, 451) | 5.35 (5.02, 5.73) | 5.52 (5.11, 6.02) |
| Fall 5 | 268 | 208 | 811 (611, 1127) | 553 (417, 778) | 15.64 (14.80, 16.58) | 15.89 (14.87, 17.05) |
| Winter 1 | 202 | 123 | 452 (354, 614) | 285 (210, 426) | 1.90 (1.83, 1.97) | 1.95 (1.85, 2.06) |
| Winter 2 | 174 | 103 | 336 (271, 445) | 222 (164, 335) | 4.66 (4.50, 4.82) | 4.68 (4.46, 4.93) |
| Winter 3 | 98 | 57 | 350 (212, 654) | 112 (79, 195) | 1.59 (1.54, 1.63) | 1.62 (1.55, 1.70) |
| Winter 4 | 145 | 80 | 468 (318, 748) | 218 (143, 382) | 1.47 (1.43, 1.51) | 1.48 (1.42, 1.54) |
| Winter 5 | 236 | 112 | 584 (454, 791) | 358 (232, 613) | 2.02 (1.96, 2.09) | 2.0 (1.89, 2.11) |
| Lab 1 | 43 | 39 | 82 (57, 154) | 65 (48, 115) | 1.77 (1.69, 1.85) | 1.79 (1.7, 1.88) |
| Lab 2 | 46 | 46 | 104 (68, 200) | 104 (68, 200) | 1.5 (1.44, 1.57) | 1.5 (1.44, 1.57) |
| **III. OTUs formed at 0.10 distance^c^** |  |  |  |  |  |  |
| **Blade**  **ID** | **Observed**  **OTUs** | **Observed OTUs**  **(Normalized subsample)** | **Chao1** | **Chao1**  **(Normalized subsample)** | **Inverse Simpson**  **Index** | **Inverse Simpson Index (Normalized subsample)** |
| Fall 1 | 74 | 60 | 262 (151, 532) | 186 (107, 395) | 3.74 (3.53, 3.98) | 3.78 (3.53, 4.05) |
| Fall 2 | 69 | 47 | 126 (94, 199) | 110 (70, 220) | 1.61 (1.56, 1.66) | 1.57 (1.5, 1.63) |
| Fall 3 | 95 | 82 | 267 (177, 456) | 220 (145, 384) | 2.16 (2.06, 2.27) | 2.15 (2.04, 2.28) |
| Fall 4 | 53 | 46 | 100 (71, 178) | 89 (61, 165) | 2.15 (2.07, 2.25) | 2.16 (2.06, 2.28) |
| Fall 5 | 78 | 69 | 153 (112, 247) | 131 (96, 211) | 2.52 (2.41, 2.65) | 2.58 (2.44, 2.75) |
| Winter 1 | 69 | 43 | 131 (96, 211) | 108 (66, 230) | 1.65 (1.6, 1.7) | 1.7 (1.63, 1.78) |
| Winter 2 | 48 | 34 | 106 (69, 210) | 55 (41, 99) | 3.08 (3.02, 3.1) | 3.04 (2.95, 3.13) |
| Winter 3 | 32 | 17 | 137 (63, 382) | 24 (18, 54) | 1.35 (1.32, 1.38) | 1.32 (1.28, 1.37) |
| Winter 4 | 44 | 31 | 220 (100, 597) | 78 (46, 182) | 1.27 (1.25, 1.30) | 1.28 (1.24, 1.32) |
| Winter 5 | 61 | 39 | 141 (92, 256) | 56 (45,92) | 1.58 (1.54, 1.62) | 1.58 (1.51, 1.65) |
| Lab 1 | 21 | 21 | 32 (24, 69) | 39 (25, 96) | 1.70 (1.64, 1.78) | 1.68 (1.61, 1.76) |
| Lab 2 | 24 | 24 | 63 (34, 175) | 63 (34, 175) | 1.46 (1.41, 152 | 1.46 (1.41, 1.52) |

^a^(=“species”, 97% similarity)

^b^(=“genus”, 95% similarity)

^c^(=“family”, 90% similarity)

Table S5. Analysis of Molecular Variance comparing Fall (n=5), Winter (n=5) and Laboratory (n=2) samples from two different distance matrices. An experiment-wise error rate of 0.0125 was used in each experiment to test for significance.

| **A. Distance matrix based upon Yue-Clayton theta** |  |  |  |
| --- | --- | --- | --- |
| **1. F-L-W- Group Test** |  |  |  |
|  | **Among** | **Within** | **Total** |
| SS | 1.83004 | 0.989276 | 2.81932 |
| df | 2 | 9 | 11 |
| MS | 0.915021 | 0.10992 |  |
| Fs: | 8.32446; *p*-value: <0.001* |  |  |
| **2. Pair-wise tests** |  |  |  |
| **F-L** | **Among** | **Within** | **Total** |
| SS | 1.16767 | 0.830391 | 1.99806 |
| df | 1 | 5 | 6 |
| MS | 1.16767 | 0.166078 |  |
| Fs: | 7.03082; *p*-value: <0.001* |  |  |
| **F-W** | **Among** | **Within** | **Total** |
| SS | 0.438612 | 0.988493 | 1.42711 |
| df | 1 | 8 | 9 |
| MS | 0.438612 | 0.123562 |  |
| Fs: | 3.54975; *p*-value: 0.007* |  |  |
| **L-W** | **Among** | **Within** | **Total** |
| SS | 1.34296 | 0.159668 | 1.50263 |
| df | 1 | 5 | 6 |
| MS | 1.34296 | 0.0319337 |  |
| Fs: | 42.0547; *p*-value: <0.001* |  |  |
| **B. Distance matrix based upon Jaccard similarity coefficient** |  |  |  |
| **1. F-L-W Group Tests** |  |  |  |
|  | **Among** | **Within** | **Total** |
| SS | 1.21479 | 3.46564 | 4.68043 |
| df | 2 | 9 | 11 |
| MS | 0.607394 | 0.385071 |  |
| Fs: | 1.57736; *p*-value: <0.001* |  |  |
| **2. Pair-wise tests** |  |  |  |
| **F-L** | **Among** | **Within** | **Total** |
| SS | 0.701448 | 1.83115 | 2.5326 |
| df | 1 | 5 | 6 |
| MS | 0.701448 | 0.36623 |  |
| Fs: | 1.91532; *p*-value: <0.001* |  |  |
| **F-W** | **Among** | **Within** | **Total** |
| SS | 0.532765 | 3.10968 | 3.64245 |
| df | 1 | 8 | 9 |
| MS | 0.532765 | 0.388711 |  |
| Fs: | 1.3706; *p*-value: <0.001* |  |  |
| **L-W** | **Among** | **Within** | **Total** |
| SS | 0.619954 | 1.99045 | 2.6104 |
| df | 1 | 5 | 6 |
| MS | 0.619954 | 0.39809 |  |
| Fs: | 1.55732; *p*-value: <0.001* |  |  |

**Table S6. Sequence classification of OTUs (0.03 distance) shared among V8 fall and winter**

| Tax Level | Taxon | Total seqs |
| --- | --- | --- |
| 1 | Bacteria | 2890 |
| 2 | Actinobacteria | 78 |
| 3 | Actinobacteria | 78 |
| 4 | Acidimicrobiales | 15 |
| 5 | Acidimicrobiaceae | 4 |
| 6 | *Ilumatobacter* | 4 |
| 5 | Iamiaceae | 10 |
| 6 | *Iamia* | 10 |
| 5 | unclassified | 1 |
| 6 | unclassified | 1 |
| 4 | unclassified | 63 |
| 5 | unclassified | 63 |
| 6 | unclassified | 63 |
| 2 | Bacteroidetes | 1451 |
| 3 | Sphingobacteria | 1223 |
| 4 | Sphingobacteriales | 1223 |
| 5 | *Saprospiraceae* | 747 |
| 6 | *Aureispira* | 19 |
| 6 | *Haliscomenobacter* | 18 |
| 6 | *Lewinella* | 275 |
| 6 | unclassified | 435 |
| 5 | Chitinophagaceae | 19 |
| 6 | unclassified | 19 |
| 5 | unclassified | 457 |
| 6 | unclassified | 457 |
| 3 | Flavobacteria | 33 |
| 4 | Flavobacteriales | 33 |
| 5 | Flavobacteriaceae | 33 |
| 6 | Tenacibaculum | 6 |
| 6 | unclassified | 27 |
| 3 | unclassified | 195 |
| 4 | unclassified | 195 |
| 5 | unclassified | 195 |
| 6 | unclassified | 195 |
| 2 | Chloroflexi | 47 |
| 3 | unclassified | 47 |
| 4 | unclassified | 47 |
| 5 | unclassified | 47 |
| 6 | unclassified | 47 |
| 2 | Planctomycetes | 29 |
| 3 | Planctomycetacia | 29 |
| 4 | Planctomycetales | 29 |
| 5 | Planctomycetaceae | 29 |
| 6 | *Rhodopirellula* | 23 |
| 6 | unclassified | 6 |
| 2 | Proteobacteria | 589 |
| 3 | Alphaproteobacteria | 480 |
| 4 | Caulobacterales | 52 |
| 5 | Hyphomonadaceae | 33 |
| 6 | *Hellea* | 12 |
| 6 | unclassified | 21 |
| 5 | unclassified | 19 |
| 6 | unclassified | 19 |
| 4 | Rhizobiales | 1 |
| 5 | unclassified | 1 |
| 6 | unclassified | 1 |
| 4 | Rhodobacterales | 4 |
| 5 | Rhodobacteraceae | 4 |
| 6 | Loktanella | 1 |
| 6 | unclassified | 3 |
| 4 | unclassified | 423 |
| 5 | unclassified | 423 |
| 6 | unclassified | 423 |
| 3 | Gammaproteobacteria | 91 |
| 4 | Alteromonadales | 3 |
| 5 | Pseudoalteromonadaceae | 3 |
| 6 | Pseudoalteromonas | 3 |
| 4 | Chromatiales | 33 |
| 5 | Granulosicoccaceae | 25 |
| 6 | *Granulosicoccus* | 25 |
| 5 | unclassified | 8 |
| 6 | unclassified | 8 |
| 4 | unclassified | 55 |
| 5 | unclassified | 55 |
| 6 | unclassified | 55 |
| 3 | unclassified | 18 |
| 4 | unclassified | 18 |
| 5 | unclassified | 18 |
| 6 | unclassified | 18 |
| 2 | unclassified | 696 |
| 3 | unclassified | 696 |
| 4 | unclassified | 696 |
| 5 | unclassified | 696 |
| 6 | unclassified | 696 |

Table S7. These V8 OTUs are found at ≥ 1% in at least one field sample (n=10) or lab sample (n=2). Recovery of sequences by F, L, W and bias is shown.

| A. Field Blades |  |  |  |  |  |
| --- | --- | --- | --- | --- | --- |
| **OTU ID** | **Taxonomic classification** | **Fall total seqs** | **Lab total seqs** | **Winter total seqs** | **Bias** |
| 1398 | Bacteroidetes"(97);"Sphingobacteria"(96);"Sphingobacteriales"(96);unclassified;unclassified; | 62 | 0 | 1 | F |
| 1442 | Bacteroidetes"(99);"Sphingobacteria"(99);"Sphingobacteriales"(99);"Saprospiraceae"(94);unclassified(93); | 92 | 0 | 7 | F |
| 1515 | Bacteroidetes"(96);"Sphingobacteria"(92);"Sphingobacteriales"(92);unclassified(82);unclassified(82); | 48 | 0 | 2 | F |
| 1521 | Bacteroidetes"(100);"Sphingobacteria"(99);"Sphingobacteriales"(99);unclassified(95);unclassified(95); | 476 | 0 | 15 | F |
| 1558 | Bacteroidetes"(100);"Sphingobacteria"(100);"Sphingobacteriales"(100);"Saprospiraceae"(100);*Lewinella(*100); | 76 | 0 | 2 | F |
| 1875 | Bacteroidetes"(98);"Sphingobacteria"(98);"Sphingobacteriales"(98);unclassified(96);unclassified(96); | 277 | 0 | 5 | F |
| 1883 | Bacteroidetes"(99);"Sphingobacteria"(98);"Sphingobacteriales"(98);unclassified(98);unclassified(98); | 88 | 0 | 5 | F |
| 1982 | Bacteroidetes"(96);"Sphingobacteria"(93);"Sphingobacteriales"(93);"Saprospiraceae"(84);unclassified; | 115 | 1 | 3 | F |
| 2302 | Bacteroidetes"(100);"Sphingobacteria"(100);"Sphingobacteriales"(100);"Saprospiraceae"(100);*Lewinella*(100); | 223 | 0 | 21 | F |
| 2381 | Bacteroidetes"(95);"Sphingobacteria"(93);"Sphingobacteriales"(93);unclassified(82);unclassified(82); | 221 | 0 | 104 | E |
| 2383 | Bacteroidetes"(94);"Sphingobacteria"(91);"Sphingobacteriales"(91);unclassified(87);unclassified(87); | 381 | 0 | 103 | F |
| 2410 | Bacteroidetes"(100);Flavobacteria(100);"Flavobacteriales"(100);Flavobacteriaceae(100);unclassified(100); | 471 | 1 | 55 | F |
| 2434 | Bacteroidetes"(97);"Sphingobacteria"(97);"Sphingobacteriales"(97);"Saprospiraceae"(87);unclassified(87); | 490 | 2 | 408 | E |
| 2435 | Bacteroidetes"(91);unclassified(83);unclassified(83);unclassified(83);unclassified(83); | 411 | 0 | 28 | F |
| 2458 | Bacteroidetes"(100);"Sphingobacteria"(100);"Sphingobacteriales"(100);"Saprospiraceae"(100);unclassified(100); | 124 | 0 | 29 | F |
| 2461 | Bacteroidetes"(100);"Sphingobacteria"(89);"Sphingobacteriales"(89);unclassified;unclassified; | 81 | 0 | 8 | F |
| 2475 | Bacteroidetes"(100);"Sphingobacteria"(100);"Sphingobacteriales"(100);"Saprospiraceae"(100);*Lewinella*(100); | 120 | 0 | 752 | W |
| 2497 | Bacteroidetes"(97);"Sphingobacteria"(97);"Sphingobacteriales"(97);"Saprospiraceae"(83);unclassified(82); | 219 | 0 | 107 | E |
| 2521 | Bacteroidetes"(100);"Sphingobacteria"(95);"Sphingobacteriales"(95);"Saprospiraceae"(92);unclassified(92); | 4817 | 65 | 15369 | W |
| 2525 | Bacteroidetes"(99);"Sphingobacteria"(99);"Sphingobacteriales"(99);"Saprospiraceae"(92);unclassified(92); | 304 | 1 | 122 | F |
| 2493 | Chloroflexi"(99);unclassified(99);unclassified(99);unclassified(99);unclassified(99); | 113 | 2 | 193 | F |
| 2468 | Chloroflexi"(100);unclassified(100);unclassified(100);unclassified(100);unclassified(100); | 58 | 0 | 70 | E |
| 1940 | Planctomycetes"(99);"Planctomycetacia"(99);Planctomycetales(99);Planctomycetaceae(99);*Rhodopirellula*(92); | 68 | 0 | 3 | F |
| 2043 | Planctomycetes"(100);"Planctomycetacia"(100);Planctomycetales(100);Planctomycetaceae(100)*;Rhodopirellula*(96); | 63 | 0 | 3 | F |
| 1768 | Proteobacteria"(100);Alphaproteobacteria(100);Caulobacterales(80);Hyphomonadaceae(80);unclassified; | 2 | 0 | 53 | W |
| 2219 | Proteobacteria"(100);Alphaproteobacteria(100);unclassified(100);unclassified(100);unclassified(100); | 27 | 1 | 502 | W |
| 2231 | Proteobacteria"(100);Alphaproteobacteria(100);unclassified;unclassified;unclassified; | 6 | 0 | 42 | W |
| 2394 | Proteobacteria"(100);Alphaproteobacteria(100);unclassified(99);unclassified(99);unclassified(99); | 82 | 0 | 1 | F |
| 2398 | Proteobacteria"(100);Alphaproteobacteria(100);unclassified(100);unclassified(100);unclassified(100); | 78 | 0 | 383 | W |
| 2408 | Proteobacteria"(100);Gammaproteobacteria(100);Chromatiales(100);Granulosicoccaceae(100);*Granulosicoccus*(100); | 66 | 1 | 330 | W |
| 2494 | Proteobacteria"(100);Alphaproteobacteria(100);Caulobacterales(100);Hyphomonadaceae(100);unclassified; | 53 | 65 | 361 | W |
| 2514 | Proteobacteria"(100);Alphaproteobacteria(100);unclassified(100);unclassified(100);unclassified(100); | 266 | 0 | 395 | E |
| 1147 | Bacteria(100);unclassified(100);unclassified(100);unclassified(100);unclassified(100);unclassified(100); | 48 | 0 | 0 | F |
| 2258 | Bacteria(100);unclassified(88);unclassified(88);unclassified(88);unclassified(88);unclassified(88); | 128 | 0 | 20 | F |
| 2489 | Bacteria(100);unclassified(88);unclassified(88);unclassified(88);unclassified(88);unclassified(88); | 716 | 0 | 55 | F |
| 2500 | Bacteria(100);unclassified(89);unclassified(89);unclassified(89);unclassified(89);unclassified(89); | 465 | 0 | 157 | F |
| 2524 | Bacteria(100);unclassified;unclassified;unclassified;unclassified;unclassified; | 32 | 0 | 684 | W |
| B. Lab Blades |  |  |  |  |  |
| **OTU ID** | **Taxonomic classification** | **Fall total seqs** | **Lab total seqs** | **Winter total seqs** | **Bias** |
| 2367 | “Bacteroidetes"(100); Flavobacteria(100);"Flavobacteriales(100);Flavobacteriaceae(100);unclassified(100); | 0 | 629 | 2 | W |
| 2427 | “Bacteroidetes"(100); Flavobacteria(100);"Flavobacteriales(100);Flavobacteriaceae(100);unclassified(100); | 1 | 2525 | 61 | W |
| 2521 | "Bacteroidetes"(100); "Sphingobacteria"(95); "Sphingobacteriales"(95); "Saprospiraceae"(92); unclassified (92); | 4817 | 65 | 15369 | W |
| 1352 | "Planctomycetes"(99); "Planctomycetacia"(99); Planctomycetales(99); Planctomycetaceae(99);unclassified(99) | 0 | 166 | 0 | n/a |
| 1938 | Proteobacteria"(100); Alphaproteobacteria(100); Sphingomonadales(99); Sphingomonadaceae(99); *Sphingopyxis*(98) | 0 | 109 | 8 | W |
| 2225 | "Proteobacteria"(100); Alphaproteobacteria(100); Rhodobacterales(100);Rhodobacteraceae(100);unclassified(97); | 9 | 119 | 7 | E |
| 2494 | "Proteobacteria"(100); Alphaproteobacteria(100); Caulobacteriales(100); Hyphomonadaceae(100);unclassified; | 53 | 65 | 361 | W |
| 2182 | "Proteobacteria"(100); Gammaproteobacteria(100); Alteromonadales(99); Alteromonadaceae(99); unclassified(90) | 0 | 213 | 1 | W |

Table S8. V5V6 OTUs that have ≥1% abundance on 1 or more blades. Sequence numbers given for F, L. W, and bias.

| A. Field Blades |  |  |  |  |  |
| --- | --- | --- | --- | --- | --- |
| **OTU ID** | **Taxonomic Classification** | **Fall total seqs** | **Lab total seqs** | **Winter total seqs** | **Bias** |
| 4136 | Actinobacteria"(100);Actinobacteria(100);unclassified(89);unclassified(89);unclassified(89); | 156 | 0 | 11 | F |
| 4422 | Actinobacteria"(100);Actinobacteria(100);Acidimicrobiales(84);unclassified(81);unclassified(81); | 469 | 0 | 73 | F |
| 4509 | Bacteroidetes"(100);"Sphingobacteria"(96);"Sphingobacteriales"(96);unclassified(96);unclassified (96); | 501 | 1 | 2158 | W |
| 4580 | Bacteroidetes"(100);"Sphingobacteria"(96);"Sphingobacteriales"(96);unclassified(90);unclassified (90); | 32 | 2 | 4858 | W |
| 4319 | Bacteroidetes"(100);"Sphingobacteria"(100);"Sphingobacteriales"(100);unclassified(100); unclassified(100); | 384 | 0 | 39 | F |
| 4385 | Bacteroidetes"(100);"Sphingobacteria"(80);"Sphingobacteriales"(80);"Saprospiraceae"(73); unclassified(54); | 2 | 0 | 184 | W |
| 4507 | Bacteroidetes"(100);Flavobacteria(100);"Flavobacteriales"(100);Flavobacteriaceae(100); unclassified(100); | 4547 | 1 | 1804 | E |
| 3944 | Bacteroidetes"(100);Flavobacteria(100);"Flavobacteriales"(100);Flavobacteriaceae(100); unclassified(87); | 225 | 6 | 38 | F |
| 4599 | Chloroflexi"(97);unclassified(97);unclassified(97);unclassified(97);unclassified(97); | 6908 | 107 | 35971 | W |
| 3994 | Planctomycetes"(100);"Planctomycetacia"(100);Planctomycetales(100);Planctomycetaceae(100); unclassified(100); | 649 | 2 | 99 | F |
| 3536 | Proteobacteria"(100);Alphaproteobacteria(98);unclassified(98);unclassified(98);unclassified(98); | 5 | 0 | 44 | W |
| 3563 | Proteobacteria"(100);Alphaproteobacteria(100);Caulobacterales(100);Hyphomonadaceae(100); *Hellea(*99); | 6 | 0 | 613 | W |
| 3575 | Proteobacteria"(100);Alphaproteobacteria(98);unclassified(98);unclassified(98);unclassified(98); | 20 | 0 | 148 | W |
| 3579 | Proteobacteria"(100);Alphaproteobacteria(100);unclassified(100);unclassified(100);unclassified (100); | 2 | 0 | 37 | W |
| 3589 | "Proteobacteria"(100);Alphaproteobacteria(98);unclassified(98);unclassified(98);unclassified(98); | 27 | 0 | 194 | W |
| 3606 | "Proteobacteria"(100);Alphaproteobacteria(100);unclassified(100);unclassified(100);unclassified (100); | 9 | 0 | 111 | W |
| 3610 | "Proteobacteria"(100);Alphaproteobacteria(97);unclassified(97);unclassified(97);unclassified(97); | 8 | 0 | 77 | W |
| 4381 | "Proteobacteria"(100);Alphaproteobacteria(97);unclassified(97);unclassified(97);unclassified(97); | 19 | 0 | 96 | W |
| 3603 | Bacteria(100);unclassified(100);unclassified(100);unclassified(100);unclassified(100);unclassified(100); | 0 | 0 | 67 | W |
| 3867 | Bacteria(100);unclassified(100);unclassified(100);unclassified(100);unclassified(100);unclassified(100); | 647 | 0 | 15 | F |
| 4252 | Bacteria(100);unclassified(100);unclassified(100);unclassified(100);unclassified(100);unclassified(100); | 4188 | 1 | 33 | F |
| 4392 | Bacteria(100);unclassified(100);unclassified(100);unclassified(100);unclassified(100); unclassified(100); | 10517 | 7 | 224 | F |
| 4491 | Bacteria(100);unclassified(100);unclassified(100);unclassified(100);unclassified(100); unclassified(100); | 1983 | 3 | 1612 | E |
| B. Lab Blades |  |  |  |  |  |
| **OTU ID** | **Taxonomic classification** | **Fall total seqs** | **Lab total seqs** | **Winter total seqs** | **Bias** |
| 2380 | Bacteria(100);"Bacteroidetes"(100);Flavobacteria(100);"Flavobacteriales"(100);Flavobacteriaceae(100); unclassified(100); | 0 | 124 | 0 | n/a |
| 2383 | Bacteria(100);"Bacteroidetes"(100);Flavobacteria(100);"Flavobacteriales"(100);Flavobacteriaceae(100); unclassified(100); | 0 | 134 | 0 | n/a |
| 4510 | Bacteria(100);"Bacteroidetes"(100);Flavobacteria(100);"Flavobacteriales"(100);Flavobacteriaceae(100); unclassified(100); | 7 | 10416 | 66 | W |
| 2404 | Bacteria(100);"Proteobacteria"(100);Alphaproteobacteria(100);Caulobacterales(83);Hyphomonadaceae(83); unclassified(83); | 0 | 156 | 0 | n/a |
| 2417 | Bacteria(100);"Proteobacteria"(100);Alphaproteobacteria(100);unclassified(97);unclassified(97);unclassified(97); | 0 | 136 | 0 | n/a |
| 3958 | Bacteria(100);"Proteobacteria"(100);Alphaproteobacteria(100);Rhodobacterales(100);Rhodobacteraceae(100); unclassified(100); | 0 | 104 | 1 | rare |
| 4349 | Bacteria(100);"Proteobacteria"(100);Alphaproteobacteria(100);Rhodobacterales(100);Rhodobacteraceae(100); unclassified(100); | 4 | 674 | 14 | W |
| 2388 | Bacteria(100);unclassified(98);unclassified(98);unclassified(98);unclassified(98);unclassified(98); | 0 | 134 | 0 | n/a |
| 2443 | Bacteria(100);unclassified(86);unclassified(86);unclassified(86);unclassified(86);unclassified(86); | 0 | 390 | 0 | n/a |
| 2448 | Bacteria(100);unclassified(100);unclassified(100);unclassified(100);unclassified(100);unclassified(100); | 0 | 381 | 0 | n/a |

Table S9. OTUs significantly associated (*p*<0.05) with at least one axis, in descending order of effect, with number in Fig S3 and taxonomic classification shown. Bias refers to sequence distribution between F, W, L pooled samples; Fw indicates that most sequences were from fall, but a sequences were found in the winter group.

| **OTU** | **Fig S3** | **Bias** |  |
| --- | --- | --- | --- |
| Otu2521 | 1 | W(Fl) | "Bacteroidetes"(100);"Sphingobacteria"(95);"Sphingobacteriales"(95);"Saprospiraceae"(92);unclassified(92); |
| Otu1305 | 2 | L | "Bacteroidetes"(100);Flavobacteria(100);"Flavobacteriales"(100);Flavobacteriaceae(100);unclassified(100); |
| Otu1311 | 3 | L | Bacteria(100);unclassified |
| Otu1322 | 4 | L | ;"Bacteroidetes"(100);Flavobacteria(100);"Flavobacteriales"(100);Flavobacteriaceae(100);unclassified(100); |
| Otu1303 | 5 | L | Bacteria(100);unclassified |
| Otu1308 | 6 | L | ;"Bacteroidetes"(100);Flavobacteria(100);"Flavobacteriales"(100);Flavobacteriaceae(100);unclassified(100); |
| Otu1328 | 7 | L | ;"Bacteroidetes"(100);Flavobacteria(100);"Flavobacteriales"(100);Flavobacteriaceae(100);unclassified(100); |
| Otu1338 | 8 | L | ;"Proteobacteria"(100);Gammaproteobacteria(100);Alteromonadales(100);Alteromonadaceae(100);unclassified; |
| Otu1348 | 9 | L | "Proteobacteria"(100);Alphaproteobacteria(100);Sphingomonadales(80);unclassified;unclassified; |
| Otu1297 | 10 | L | "Bacteroidetes"(100);Flavobacteria(100);"Flavobacteriales"(100);Flavobacteriaceae(100);unclassified(80); |
| Otu1316 | 11 | L | "Proteobacteria"(100);Gammaproteobacteria(100);Pseudomonadales(100);Pseudomonadaceae(100);unclassified(100); |
| Otu1318 | 12 | L | "Proteobacteria"(100);Alphaproteobacteria(100);Rhodobacterales(100);Rhodobacteraceae(100);unclassified(100); |
| Otu1352 | 13 | L | "Planctomycetes"(99);"Planctomycetacia"(99);Planctomycetales(99);Planctomycetaceae(99);unclassified(99); |
| Otu1991 | 14 | F | "Bacteroidetes"(100);Flavobacteria(100);"Flavobacteriales"(100);Flavobacteriaceae(100);unclassified(100); |
| Otu2182 | 15 | L | "Proteobacteria"(100);Gammaproteobacteria(100);Alteromonadales(99);Alteromonadaceae(99);unclassified(90); |
| Otu0994 | 16 | F | "Bacteroidetes"(100);"Sphingobacteria"(100);"Sphingobacteriales"(100);"Saprospiraceae"(96);unclassified(92); |
| Otu1213 | 17 | F | Bacteria(100);unclassified;unclassified;unclassified;unclassified;unclassified; |
| Otu1196 | 18 | F | "Bacteroidetes"(100);"Sphingobacteria"(98);"Sphingobacteriales"(98);"Saprospiraceae"(89);unclassified; |
| Otu0941 | 19 | F | "Actinobacteria"(100);Actinobacteria(100);Acidimicrobiales(88);unclassified;unclassified; |
| Otu2524 | 20 | Wf | Bacteria(100);unclassified;unclassified;unclassified;unclassified;unclassified; |
| Otu2491 | 21 | W | Bacteria(100);unclassified(82);unclassified(82);unclassified(82);unclassified(82);unclassified(82); |
| Otu2367 | 22 | L(w) | "Bacteroidetes"(100);Flavobacteria(100);"Flavobacteriales"(100);Flavobacteriaceae(100);unclassified(100); |
| Otu2350 | 23 | W(f) | "Bacteroidetes"(85);"Sphingobacteria"(85);"Sphingobacteriales"(85);unclassified;unclassified; |
| Otu1226 | 24 | F | "Bacteroidetes"(88);"Sphingobacteria"(88);"Sphingobacteriales"(88);unclassified;unclassified; |
| Otu0489 | 25 | F | "Bacteroidetes"(93);unclassified;unclassified;unclassified;unclassified; |
| Otu1150 | 26 | F | "Bacteroidetes"(100);"Sphingobacteria"(100);"Sphingobacteriales"(100);"Saprospiraceae"(80);unclassified(80); |
| Otu1723 | 27 | F | "Bacteroidetes"(100);"Sphingobacteria"(97);"Sphingobacteriales"(97);"Saprospiraceae"(97);unclassified(85); |
| Otu2426 | 28 | Fw | "Bacteroidetes"(97);"Sphingobacteria"(97);"Sphingobacteriales"(97);"Saprospiraceae"(89);unclassified(89); |
| Otu1442 | 29 | F | "Bacteroidetes"(99);"Sphingobacteria"(99);"Sphingobacteriales"(99);"Saprospiraceae"(94);unclassified(93); |
| Otu2408 | 30 | Wf(l) | "Proteobacteria"(100);Gammaproteobacteria(100);Chromatiales(100);Granulosicoccaceae(100);Granulosicoccus(100); |
| Otu2067 | 31 | W | Bacteria(100);unclassified(100) |
| Otu2302 | 32 | F(w) | "Bacteroidetes"(100);"Sphingobacteria"(100);"Sphingobacteriales"(100);"Saprospiraceae"(100);Lewinella(100); |
| Otu2016 | 33 | F | "Proteobacteria"(100);Gammaproteobacteria(100);unclassified(100);unclassified(100);unclassified(100); |
| Otu1001 | 34 | F | "Bacteroidetes"(100);"Sphingobacteria"(98);"Sphingobacteriales"(98);"Saprospiraceae"(98);Lewinella(95); |
| Otu2225 | 35 | L(fw) | "Proteobacteria"(100);Alphaproteobacteria(100);Rhodobacterales(100);Rhodobacteraceae(100);unclassified(97); |
| Otu1521 | 36 | F | "Bacteroidetes"(100);"Sphingobacteria"(99);"Sphingobacteriales"(99);unclassified(95);unclassified(95); |
| Otu1875 | 37 | F | "Bacteroidetes"(98);"Sphingobacteria"(98);"Sphingobacteriales"(98);unclassified(96);unclassified(96); |
| Otu1230 | 38 | F | Bacteria (100);unclassified(85) |
| Otu1982 | 39 | F | "Bacteroidetes"(96);"Sphingobacteria"(93);"Sphingobacteriales"(93);"Saprospiraceae"(84);unclassified; |
| Otu2435 | 40 | F(w) | "Bacteroidetes"(91);unclassified(83);unclassified(83);unclassified(83);unclassified(83); |
| Otu1121 | 41 | F | "Bacteroidetes"(80);"Sphingobacteria"(80);"Sphingobacteriales"(80);unclassified(80);unclassified(80); |
| Otu1237 | 42 | F | Bacteria(100); unclassified |
| Otu2071 | 43 | F | "Bacteroidetes"(100);Flavobacteria(100);"Flavobacteriales"(100);Flavobacteriaceae(100);Tenacibaculum(80); |
| Otu2393 | 44 | F | "Proteobacteria"(100);Gammaproteobacteria(98);unclassified(98);unclassified(98);unclassified(98); |
| Otu1558 | 45 | F | "Bacteroidetes"(100);"Sphingobacteria"(100);"Sphingobacteriales"(100);"Saprospiraceae"(100);Lewinella(100); |
| Otu0984 | 46 | F | "Proteobacteria"(100);Alphaproteobacteria(100);unclassified(100);unclassified(100);unclassified(100); |
| Otu0844 | 47 | F | "Bacteroidetes"(100);"Sphingobacteria"(100);"Sphingobacteriales"(100);"Saprospiraceae"(100);unclassified(100); |
| Otu2178 | 48 | Wf | "Proteobacteria"(100);Alphaproteobacteria(100);unclassified(100);unclassified(100);unclassified(100); |
| Otu2518 | 49 | F | Bacteria(100);unclassified(100);unclassified(100);unclassified(100);unclassified(100);unclassified(100); |
| Otu1938 | 50 | Lw | "Proteobacteria"(100);Alphaproteobacteria(100);Sphingomonadales(99);Sphingomonadaceae(99);Sphingopyxis(98); |
| Otu2471 | 51 | F | "Bacteroidetes"(82);unclassified;unclassified;unclassified;unclassified; |
| Otu2410 | 52 | F(w) | "Bacteroidetes"(100);Flavobacteria(100);"Flavobacteriales"(100);Flavobacteriaceae(100);unclassified(100); |
| Otu2461 | 53 | F | "Bacteroidetes"(100);"Sphingobacteria"(89);"Sphingobacteriales"(89);unclassified;unclassified; |
| Otu1050 | 54 | F | "Bacteroidetes"(82);"Sphingobacteria"(82);"Sphingobacteriales"(82);unclassified;unclassified; |
| Otu2398 | 55 | WF | "Proteobacteria"(100);Alphaproteobacteria(100);unclassified(100);unclassified(100);unclassified(100); |
| Otu2500 | 56 | Wf | Bacteria(100);unclassified(89) |
| Otu2489 | 57 | Fw | Bacteria(100);unclassified(88);unclassified(88);unclassified(88);unclassified(88);unclassified(88); |
| Otu1763 | 58 | Fw | "Proteobacteria"(98);Alphaproteobacteria(98);unclassified(98);unclassified(98);unclassified(98); |
| Otu2332 | 59 | W | "Bacteroidetes"(100);"Sphingobacteria"(100);"Sphingobacteriales"(100);"Saprospiraceae"(90);unclassified; |
| Otu2427 | 60 | Lw | "Bacteroidetes"(100);Flavobacteria(100);"Flavobacteriales"(100);Flavobacteriaceae(100);unclassified(100); |
| Otu2381 | 61 | Fw | "Bacteroidetes"(95);"Sphingobacteria"(93);"Sphingobacteriales"(93);unclassified(82);unclassified(82); |
| Otu2394 | 62 | F | "Proteobacteria"(100);Alphaproteobacteria(100);unclassified(99);unclassified(99);unclassified(99); |
| Otu1527 | 63 | F | "Bacteroidetes"(97);unclassified;unclassified;unclassified;unclassified; |
| Otu1147 | 64 | F | Bacteria(100);unclassified(100);unclassified(100);unclassified(100);unclassified(100);unclassified(100); |
| Otu2525 | 65 | Fw | "Bacteroidetes"(99);"Sphingobacteria"(99);"Sphingobacteriales"(99);"Saprospiraceae"(92);unclassified(92); |
| Otu2044 | 66 | F | "Bacteroidetes"(93);"Sphingobacteria"(85);"Sphingobacteriales"(85);unclassified;unclassified; |
| Otu1674 | 67 | Fw | "Bacteroidetes"(100);"Sphingobacteria"(100);"Sphingobacteriales"(100);"Saprospiraceae"(100);Lewinella(95); |
